# Supplementary material for: Four New Species of Dictyostelids from Soil Systems in Northern Thailand
Source: J Fungi (Basel). 2022 May 31;8(6):593. doi: 10.3390/jof8060593 (PMC9225131; doi:10.3390/jof8060593)
Supplement: Supplementary file 1 [file jof-08-00593-s001.zip › jof-1696244-supplementary.pdf]

S1. Sequence alignment

>AM168070.1_Cavenderia_multistipes_UK26b
catgcatgtctaagtatag--
tcctcacggacgaaactgcagacggctcattacaacggtcgctggtcacaggacacggggcaactcgctgtggataaccgcat
taaatcggggctaatacatacaaaccgagtggacgactgggcaa-ctggaagttcgcgcgatgga---
ttaacccttcgactgacctct---tggtttggttggaaccgattcatgttgttagtcggc--------
gcttcggcgccgatttgctagacgcgactgccctatcagcttgacggcaaggtcttggcttgccgtggctgtaacgggtaacgg
agaattagggttcgattccggagaggacgcctgagaaacggcgtccacacctacgggtggcagcaggcacgtaaatttccca
atgtcaata-
cgatgagggagcgacaataaatacttttagtcctggtcgcaagatcaggtcaacaagattaagcacaaagtaaataaattagcc
agtattattggagggcaagtctggtgccagcagccgcggtaattccagctccaacagtatatactaaagttgttgcagttcgttcg
tagcttaagtctgtgtaatgcttaagcgcggcgcggtagattgttctagtcggaggcgcggcgttcaactgtgcataaatcttgat
gctcaaggtgagtcaggcgtggacgggaaaacagtgcatggtattgtagaagaagacgtgcaacgtctaggttggttggttg
tccaggtaatgattaatagggaggagcgggggctgtcgtattgacgggcgagaggtgaaattcgttgaccctgtcaagacgc
acaacggcggaagcagcagccaagtgcctctccattagtcaagaacgatagtctagggatcaaagacgatcagataccgtcgt
agtctagaccataaactatgtcggctaacacttgg---
cgacgtgctctccgaggcgtctccaggagtttgtgggaaaccataagcttttggactctgggggagtatggccgcaagactg
aaacttaaaggaattgacggaagggcacaccatggcgtggagcctgcggcttaatttgactcaacacgggaaaccttaccaag
ctcagatatgtataggattgacagactaatagatttttcatgatcgcataagtagtggtgcatggccgttcttagttggtggagtga
tttgtcaggtcaattccggtaacggacgagacctccatttgctaactagtcgcggcgacgggttcgactggcgagggtcggac
ggcgcggctgcctgcgaaagg-
ctgggtaagtgcgtcgtacggttagtagcctgggctcgtcttcgcggaataaagtaacttcttagaaatacttccgacaccaagc
cggaggaagttggaggcaataacaggtctgtgatgcccttagatatcttgggccgcacgcgtgctacaatgtagacgcgaata
agctctaaaaatgcgcggcgcgaagcgtcgcggaaac-
agcaatcgtctacgtaacagggactgacggttgcaataacgatcacgaacgaggaatgccttgtaggcgcgagtcatcatctc
gcgccgaatctgtccctgccctttgtacacaccgcccgtcgctcctaccgatcggacggcgaggtgaagacgacggagggac
gcggaaagtcgtttaaatgttgtctagaggaag
>AM168092.1_Cavenderia_deminutiva_MexM19A
catgcatgtctaagtataagtccttgtacgactagactgcagacggctcattacaacagttgttgtctacaggacatgcttt--
tgcgttttggataaccgcagtaaatcggggctaatacatataaacgaaggaatgactggta----
acggaagtttctgcgatgg--atttagctattcaaccggccttg-----
tgtttgttggaaccgatccatattgctaatcggcattagtttactaatagcctgatgagctatgaacaactgccctatcaacttgatg
gtaaggtattggcttaccatggttgtaacgggtaacggggaatcagggttcgattccggagaggacgcctgagaaacggcgt
ccacatctacgggtggcagcaggcgcgtaaattacccaatctcaatagagaggaggtggcgacaataaatcctgatgcctata
ggctcacgcctagggcaattagaataagtacaaaataaatccattaaccaatgtaattggagggcaagtctggtgccagcagc
cgcggtaattccagctccaatagcgtatactaatgttgttgcagttcgctcgtagcttaatttttagcta-
atcgtggcatgcacgtgctggttg-
gtttgttggagcaagcttagtcaactgtgcataaaccttgatgctcaaggtaaagacgtaggtctgtgatactaagtgcatggtat
tgtagaacatggtaacaggtggctttgttggtgggtcactagtgcaatgattaatagggaggagcgggggccttcatattgatg
ggcgagaggtgaaattcgttgaccctatcaagatgtcctacagcgaaagcattggccaagtgcctctccattagtcaagaacga
tagtttggggatcaaagacgatcagataccgtcgtagtccaaaccataaacgatgtcgaccagtgattaggcacgtcacctttct
agagaactgcctaggaacttgtgggaaaccatgagtttttggactctgggggagtatggtcgcaaggctgaaacttaaagg
aattgacggaagggcacaccatggagtggagcctgcggcttaatttgactcaacacgggaaaacttaccaagctctgatatgat

aaggattgacagactaaaagatctttcatgatcgcataagtggtggtgcatggtcgttcttagttggtggagtgatttgtcaggtc
aattccggtaacggacgagacctcgacctgctaactagtgacaattagattgtcgatgggcgaagttagttcggcagctacttaa
-tgggcaactgttaggtagttgacgtattgattgtagcctggcaatgtagttgtt---
atattttccttcttagagggacttccagctttaagttggaggaagtccgaggcaataacaggtctgtgatgcccttagatatcttgg
gccgcacgcgtgctacaatgtaggcgctaataggcattaccctttacct-----
agaggtagaggtaacctacaatcccctacgtgatggggattgaccactgtaactgtggtcatcaacgaggaattccttgtaggc
gtgcgtcactatcgcacgccgaatctgtccctgccctctgtacacaccgcccgtcgctcctaccgatcgaacgtccaggtaaagtc
gacggaagttgatagaaagtccattgaacgtcgtttagaggaag
>AM168081.1_Cavenderia_stellata_SAB7B
catgcatgtctaagtataagctcttgtacggctagactgcagacggctcattacaacggttgtagcttccaggacatgtcgcaag
gccttctggataaccgcagtaaatcggggctaatacatataaacggaggggtagagggggcaaccttgaagcttctgcgatg
gacacttagctattcgaccaaccccgcaagggagtggttggaaccggttcatattgctaatcgactctagcttgctagtagtctga
taagctatagacaactgccctatcaacttgatggtaaggttttggcttaccatggttataacgggtaacggggaatcagggttcg
attccggagaggacgcctgagaaacggcgtccacatctacgggtggcagcaggcgcgtaaattgcccaatctcaacagaga
ggaggcggtgacaataaatcccgatggctttgggggcaaccccaggccaatcagaataagtacacattaaatcccctaaccaa
tataattggagggcaagtctggtgccagcagccgcggtaattccagctccaatagcatatactaatgttgttgcagttcgctcgta
gctcaattttgagctattacgcggctcaacactgtaggtcactggtcttagggccatcgtaatcgactgtgcataaaccttgatgct
caaggtaggcctctg-
ggggtagatacgcagtgcatggcattgtggaacaaggcatctcgcggcttagttggtgggccgcgggggcaatgattaatag
ggaggagcgggggccttcatattgcagggcgagaggtgaaattcgttgacccttgcaagatgtcctacagcgaaagcattgg
ccaagtgcctctccattagtcaagaacgaaagtttggggatcaaagacgatcagataccgtcgtagtccaaaccataaactatgt
cgaccagtgattaggcgggctaccttcttcgagagctgcctagcaacttgtgggaaaccatgagtgcttggactctgggggga
gtatggtcgcaaggctgaaacttaaaggaattgacggaagggcacaccatggagtggagcctgcggcttaatttgactcaaca
cgggaaagcttaccaagctcagatatgataaggattgacagactaaaagatctttcatgatctcataagtggtggtgcatggtcg
ttcttagttggtggagtgatttgtcaggtcaattccggtaacggacgagacctcgacctgctaactagtggggctcattggttcaa
atggcgaagcgaattttgcttttactatgggggcaaccattcatagtggggtagggagcgtattagcctgggccagtgggtttc
aaaaaattaatcttcctagaggtacttctggctctaagccagaggaagtccgaggcaataacaggtctgtgatgcccttagatat
cttgggccgcacgcgtgctacaatgtaggcgctaatgagtcaacat-
tccagcaccgcaaggtgtctggtaatcttggaatcccctgcgtgattgggcttgaccactgtaactgtggtcatcaacgaggaatt
ccttgtatgcgcgagtcactatctcgcgcagaatctgtccctgccctttgtacacaccgcccgtcgctcctaccgatcgaacgatca
ggtaaagtggacagagggcttttgaaagtccattgaacgccgtttagaggaag
>AM168089.1_Cavenderia_mexicana_MexTF4B1
catgcatgtctaagtataagctcttgtacggctagactgcagacggctcattacaacggttgtatcttccaggacatgtcgaaaga
ccttctggataaccgcagtaaatcggggctaatacatacaaacggaggggtggatagggcaaccttgaagcttctgcgatgga
cacttagctattcgaccagccccgcaagggaacggttggaaccggttcatattgctaatcgactctggcttgccacgagtctgat
aagctatagacaaccgccctatcaacttgatggtaaggttttggcttaccatggttgtaacgggtaacggggaatcagggttcga
ttccggagaggacgcctgagaaacggcgtccacatctacgggtggcagcaggcgcgtaaattgcccaatctcaacagagag
gaggcggtgacaataaatcccgatggctttgggggcaaccccaggccaatcagaataagtacacattaaatcccttaaccaata
taattggagggcaagtctggtgccagcagccgcggtaattccagctccaatagcatatactaatgttgtcgcagttgcctcgtag
cttaattttgagctattacgcggctcgacactgtaggtcgtcattgggggtagtgtcactatcgactgtgcataaaccttgatgctc
aaggtaggcctttatagggtagatacacagtgcatggcattgtggaacaaggcatctcgcggcttagttggtgggccgcggg
ggcaatgattaatagggaggagcggggaccttcatattgcagggcgagaggtgaaattcgttgacccttgcaagatgaccga
cagcgaaagcattggtcaagtgccttcccattagtcaagaacgaaagtttggggatcaaagacgatcagataccgtcgtagtcc
aaaccataaactatgtcgaccagcgattaggcgggctaccttcttcgagagctgcctagcagcttgtgggaaaccatgagtgctt

ggactctgggggagtatggtcgcaaggctgaaacttaaaggaattgacggaagggcacaccatggagtggagcctgcgg
cttaatttgactcaacacgggaaagcttaccaagctcagatatgataaggattgacagactaaaagatctttcatgatctcataagt
ggtggtgcatggtcgttcttagttggtggagtgatttgtcaggtcaattccggtaacggacgagacctcgacctgctaactagtg
gggttcattccttcgctcgatgaggcaaggtctgcttttattgcagggggtaactcttgtagtagggtaggctttgtattagtctgg
gggagtggacttccaatttataatcttcctagaggtacttctggctctaagccagaggaagtccgaggcaataacaggtctgtga
tgcccttagatatcttgggccgcacgcgtgctacaatgtaggcgctaatgagtcaacatatccagctccgtaaggagtctggtaa
tcttggaatcccctgcgtgattgggcttgactactgtaactgtggtcatcaacgaggaattccttgtatgcgtgagtcactatctcac
gcagaatctgtccctgccctttgtacacaccgcccgtcgctcctaccgatcgaacgatcaggtaaagtggacagagggttttgg
gaagtccattgaacgccgtttagaggaag
>AM168084.1_Cavenderia_bifurcata_UK5
catgcatgtctaagtataagctcttgtacggctagactgcagacggctcattacaacggttgtagcttccaggacatgtcgcaag
accttctggataaccgcagtaaatcggggctaatacatacaaacggagggatggatagggcaaccttgaagtttctgcgatgg
acacttagctattcgaccagccctgaacaggaatggttggaaccggttcatattgctaatcgactctagcttgctagcagtctgat
aagctatagacaaccgccctatcaacttgatggtaaggttttggcttaccatggttgtaacgggtaacggggaatcagggttcga
ttccggagaggacgcctgagaaacggcgtccacatctacgggtggcagcaggcgcgtaaattgcccaatctcaacagagag
gaggcggtgacaataaatcccgatggctttgggggcaaccccaggccaatcagaataagtacacattaaatcccttaaccaata
taattggagggcaagtctggtgccagcagccgcggtaattccagctccaatagcatatactaatgttgttgcagttcgctcgtag
ctcaattttgagcttttacgcggctcaacactgtaggtcatcattagggatagtgtcataatcgactgtgcataaaccttgatgctca
aggtaggcctttatagggcagatacacagtgcatggcattgtggaacaaggcatctcgcggcttagttggtgggccgcgggg
gcaatgattaatagggaggagcgggggccttcatattgcagggcgagaggtgaaattcgttgacccttgcaagatgtccgac
agcgaaagcattggccaagtgcctctccattagtcaagaacgaaagtttggggatcaaagacgatcagataccgtcgtagtcc
aaaccataaactatgtcgaccagcgattaggcgcgctaccttcttcgagagctgcctagcagcttgtgggaaaccatgagtgctt
ggactctgggggagtatggtcgcaagtctgaaacttaaaggaattgacggaagggcacaccatggagtggagcctgcgg
cttaatttgactcaacacgggaaagcttaccaagctcagatatgataaggattgacagactaaaagatctttcatgatctcataagt
ggtggtgcatggtcgttcttagttggtggagtgatttgtcaggtcaattccggtaacggacgagacctcgacctgctaactagtg
gggttcattctttcgcttgacgaggcaggttttgctttgattacagaaggggggcttttgtagtggggtagagtttgtagtagtctgg
aggagtggacttcaaaaaaataatcttcctagaggtacttctggctctaagtcagaggaagtccgaggcaataacaggtctgtg
atgcccttaaatatcttgggccgcacgcgtgctacaatgtaggcgctagaaagtcattaaaaccatctccgcaaggagtatggta
atcttataatcacctgcgtgattgggcttgaccactgtaactgtggtcatcaacgaggaattccttgtatgcgcgagtcactatctc
gcgcagaatctgtccctgccctttgtacacaccgcccgtcgctcctaccgatcgaacgatcaggtaaagtggacagagagcttttt
ggaagtccattgaacgccgtttagaggaag
>AM168072.1_Cavenderia_granulophora_CHII_4
catgcatgtctaagtataagctcttgtacggctagactgcagacggctcattacaacggttgtatcttccaggacatgtcgcaaga
tcttctggataaccgcagtaaatcggggctaatacatacaaacgaaggggcagagagggcaaccttgaagcttctgcgatgg
acacttagctattcgaccaaccccgcaagggaatggttggaaccggttcatattgctaatcgactctggtttaccatgagtc-
gataagctatagacaaccgccctatcaacttgatggtaaggttttggcttaccatggttgtaacgggtaacggggaatcagggtt
cgattccggagaggacgcctgagaaacggcgtccacatctacgggtggcagcaggcgcgtaaattgcccaatctcaacaga
gaggaggcggtgacaataaatcccgatggctttgggggcaaccccaggccaatcagaataagtacacattaaatcccttaacc
aatataattggagggcaagtctggtgccagcagccgcggtaattccagctccaatagcatatactaatgttgttgcagttcgctc
gtagcttaattttgagctattacgcggctcaacactgtaggtcattgctagcaatagcatcataatcgactgtgcataaaccttgat
gctcaaggtaggccttttttagggtagatacacagtgcatggcattgtggaacaaggcatctcgcggcttagttggtgggccgcg
gggcaatgattaatagggaggagcgggggccttcatattgcagggcgagaggtgaaattcgttgacccttgcaagatgtcc
gacagcgaaagcattggccaagtgcctccccattagtcaagaacgaaagtttggggatcaaagacgatcagataccgtcgtag
tccagaccataaactatgtcgaccagcgattaggcgggctaccttcttcgagagctgcctagcagcttgtgggaaaccatgagt

gcttggactctgggggagtatggtcgcaaggctgaaacttaaaggaattgacggaagggcacaccatggagtggagcctg
cggcttaatttgactcaacacgggaaagcttaccaagctcagatatgataaggattgacagactaaaagatctttcatgatctcat
aagtggtggtgcatggtcgttcttagttggtggagtgatttgtcaggtcaattccggtaacggacgagacctcgacctgctaact
agtgggattcattccttctctcgacgaggcaggttctgcttttattgtaaggg--
caactttgcagtagggtaggatttgtattagtctgggggagtggatttcatataattaatcttcctagaggtacttctggctctaagc
cagaggaagttcgaggcaataacaggtctgtgatgcccttagatatcttgggccgcacgcgtgctacaatgtaggcgctaatg
agtctttacatccagctccgtaaggagtctggtaatcttggaatcccctgcgtgattgggcttgactactgtaactgtggtcatcaa
cgaggaattccttgtatgcgcgagtcactatctcgcgcagaatctgtccctgccctttgtacacaccgcccgtcgctcctaccgatc
gaacgatcaggtaaagtggacagaggcatttggaaagtccattgaacgccgtttagaggaag
>AM168088.1_Cavenderia_medusoides_OH592
catgcatgtctaagtataagctcttgtacggctagactgcagacggctcattacaacggttgtatcttccagaacgtgtcgcaaga
tcttctggataaccgcagtaaatcggggctaatacatacaaacgaaggggtggagagggcaaccttgaagcttctgcgatgga
cacttagctattcgaccaaccccgcaagggaatggttggaaccggttcatattgctaatcgactctggtttaccatgagtc-
gataagctatagacaaccgccctatcaacttgatggtaaggttttggcttaccatggttgtaacgggtaacggggaatcagggtt
cgattccggagaggacgcctgagaaacggcgtccacatctacgggtggcagcaggcgcgtaaattgcccaatctcaacaga
gaggaggcggtgacaataaatcccgatggctttgggggcaaccccaggccaatcagaataagtacacattaaatcccttaacc
aatataattggagggcaagtctggtgccagcagccgcggtaattccagctccaatagcatatactaatgttgttgcagttcgctc
gtagcttaattttgagctattacgcggctcaacactgtaggtcattactggtaacagtatcataatcgactgtgcataaaccttgatg
ctcaaggtaggcctttttagggtagatacacagtgcatggcattgtggaacaaggcatctcgcggcttagttggtgggccgcgg
gggcaatgattaatagggaggagcgggggccttcatattgcagggcgagaggtgaaattcgttgacccttgcaagatgtccg
acagcgaaagcattggccaagtgcctccccattagtcaagaacgaaagtttggggatcaaagacgatcagataccgtcgtagt
ccaaaccataaactatgtcgaccagcgattaggcgggctaccttcttcgagagctgcctagcagcttgtgggaaaccatgagtg
cttggactctgggggagtatggtcgcaaggctgaaacttaaaggaattgacggaagggcacaccatggagtggagcctgc
ggcttaatttgactcaacacgggaaagcttaccaagctcagatatgataaggattgacagactaaaagatctttcatgatctcata
agtggtggtgcatggtcgttcttagttggtggagtgatttgtcaggtcaattccggtaacggacgagacctcgacctgctaacta
gtgggattcattccttcgctcgacgaggcaggttctgcttttattgtagggg--
caactttacagtagggtaggatttgtattagtctgggggagtggatttcatataattaatcttcctaaaggtacttctggctctaagc
cagaggaagttcgaggcaataacaggtctgtgatgcccttagatatcttgggccgcacgcgtgctacaatgtaggcgctaatg
agtctttacatccagctccgtaaggagtctggtaatcttggaatcccctgcgtgattgggcttgactactgtaactgtggtcatcaa
cgaggaattccttgtatgcgcgagtcactatctcgcgcagaatctgtccctgccctttgtacacaccgcccgtcgctcctaccgatc
gaacgatcaggtaaagtggacagaggtttttggaaagtccattgaacgccgtttagaggaag
>AM168083.1_Cavenderia_aureostipes_YA6
catgcatgtctaagtataagctcttgtacggctagactgcagacggctcattacaacggttgtatcttccaggacatgtcgcaaga
ccttctggataaccgcagtaaatcggggctaatacatacaaacggaggggtagagagggcaaccttgaagcttctgcgatgg
acaattagttattcgaccaaccccgcaagggaatggttggaaccggttcatattgctaatcgactctagcttgctagtagtctgata
agctatagacaaccgccctatcaacttgatggtaaggttttggcttaccatggttgtaacgggtaacggggaatcagggttcgat
tccggagaggacgcctgagaaacggcgtccacatctacgggtggcagcaggcgcgtaaattgcccaatctcaacagagagg
aggcggcgacaataaatcccgatggctttgggggcaaccccaggccaatcagaataagtacacattaaatcccttaaccaatat
aattggagggcaagtctggtgccagcagccgcggtaattccagctccaatagcatatactaatgttgttgcagttcgctcgtagc
tcaattttgagc-
gtttcgcggctcaacactgtaggtcatagttagcaatagcttcataatcgactgtgcataaaccttgatgctcaaggtaggccttta
tagggtagatacacagtgcatggcattgtggaacaaggcatctcgcggcttagttggtgggccgcgggggcaatgattaata
gggaggagcgggggccttcatattgcagggcgagaggtgaaattcgttgacccttgcaagatgtcctacagcgaaagcattg
gccaagtgcctccccattagtcaagaacgaaagtttggggatcaaagacgatcagataccgtcgtagtccaaaccataaactat

gtcgaccagcgattaggcgggctaccttcttcgagagctgcctagcagcttgtgggaaaccatgagtgcttggactctgggggg
gagtatggtcgcaaggctgaaacttaaaggaattgacggaagggcacaccatggagtggagcctgcggcttaatttgactca
acacgggaaagcttaccaagctcagatatgattaggattgacagactaaaagatctttcatgatctcataagtggtggtgcatgg
tcgttcttagttggtggagtgatttgtcaggtcaattccggtaacggacgagacctcgacctgctaactagtgggattcattctttc
gattgacgaggcaggttttgctttgattatagggggcaacttctatagtcgggtagagcttgtattagtctgggagagtgggtttc
aaaaaattaatcttcctagaggtacttctggctctaagccagaggaagtccgaggcaataacaggtctgtgatgcccttagatat
cttgggccgcacgcgtgctacaatgtaggcgctaatgagtttttacatccatctccgcaaggagtatggtaatcttggaatcccct
gcgtgattgggcttgaccactgtaactgtggtcatcaacgaggaattccttgtatgcgcgagtcactatctcgcgcagaatctgtc
cctgccctttgtacacaccgcccgtcgctcctaccgatcgaacgatcaggtaaagtggacagagagttcttggaagtccattgaa
cgccgtttagaggaag
>HQ141522.1_Cavenderia_myxobasis_NT2A
catgcatgtctaagtataagctcttgtacggctagactgcagacggctcattacaacggttgtatcttccagggcatgtcgcaaga
ccttctggataaccgcagtaaatcggggctaatacatacaaacggagggatggagagggcaaccttgaagtttctgcgatgga
caattagttattctaccaaccccgcaagggaatggttggaaccggttcatattgctaatcgactctagcttgctagtagtctgataa
gctatagacaaccgccctatcaacttgatggtaaggttttggcttaccatggttgtaacgggtaacggggaatcagggttcgatt
ccggagaggacgcctgagaaacggcgtccacatctacgggtggcagcaggcgcgtaaattgcccaatctcaacagagagg
aggcggtgacaataaatcccgatggctttgggggcaaccccaggctaatcagaataagtacacattaaatcccttaaccaatat
aattggagggcaagtctggtgccagcagccgcggtaattccagctccaatagcatatactaatgttgttgcagttcgctcgtagc
ttaattttgagctatatcgcggctcaacactgtaggtcatggctagcaatagttgcataatcgactgtgcataaaccttgatgctca
aggtaggcctttatagggtagatacacagtgcatggcattgtggaacaaggcatctcgcggcttagttggtgggccgcgggg
gcaatgattaatagggaggagcgggggccttcatattgcagggcgagaggtgaaattcgttgacccttgcaagatgtcctaca
gcgaaagcattggccaagtgcctccccattagtcaagaacgaaagtttggggatcaaagacgatcagataccgtcgtagtcca
aaccataaactatgtcgaccagcgattaggcgggctaccttcttcgagagctgcctagcagcttgtgggaaaccatgagtgctt
ggactctgggggggagtatggtcgcaaggctgaaacttaaaggaattgacggaagggcacaccatggagtggagcctgcgg
cttaatttgactcaacacgggaaagcttaccaagctcagatatgattaggattgacagactaaaagatctttcatgatctcataagt
ggtggtgcatggtcgttcttagttggtggagtgatttgtcaggtcaattccggtaacggacgagacctcgacctgctaactagtg
ggattcattctttcgattgacgaggcaggtattgctttgattatagggggcaacttctatagtcgggtagtgcttgtagtagtctgg
aggagtggatttcaaaaaattaatcttcctagaggtacttctggctctaagccagaggaagtccgaggcaataacaggtctgtg
atgcccttagatatcttgggccgcacgcgtgctacaatgtaggcgctaatgagtttttacatccatctccgcaaggagtatggtaa
tcttggaatcacctgcgtgattgggcttgaccactgtaactgtggtcatcaacgaggaattccttgtatgcgcgagtcactatctcg
cgcagaatctgtccctgccctttgtacacaccgcccgtcgctcctaccgatcgaacgatcaggtaaagtggacagagggtacttg
gaagtccattgaacgccgtttagaggaag
>HQ141515.1_Cavenderia_subdiscoidea_TH1A
catgcatgtctaagtataagctcttgtacggctagactgcagacggctcattacaacggttgtatcttccagggcatgtcgcaaga
ccttctggataaccgcagtaaatcggggctaatacatacaaacggagggatggagagggcaaccttgaagtttctgcgatgga
caattagttattcgaccaaccccgcaagggaatggttggaaccggttcatattgctaatcgactctagcttgctagtagtctgataa
gctatagacaaccgccctatcaacttgatggtaaggttttggcttaccatggttgtaacgggtaacggggaatcagggttcgatt
ccggagaggacgcctgagaaacggcgtccacatctacgggtggcagcaggcgcgtaaattgcccaatctcaacagagagg
aggcggtgacaataaatcccgatggctttgggggcaaccccaggctaatcagaataagtacacattaaatcccttaaccaatat
aattggagggcaagtctggtgccagcagccgcggtaattccagctccaatagcatatactaatgttgttgcagttcgctcgtagc
ttaattttgagctatatcgcggctcaacactgtaggtcatggctagcaatagttgcataatcgactgtgcataaaccttgatgctca
aggtaggcctttatagggtagatacacagtgcatggcattgtggaacaaggcatctcgcggcttagttggtgggccgcgggg
gcaatgattaatagggaggagcgggggccttcatattgcagggcgagaggtgaaattcgttgacccttgcaagatgtcctaca
gcgaaagcattggccaagtgcctccccattagtcaagaacgaaagtttggggatcaaagacgatcagataccgtcgtagtcca

aaccataaactatgtcgaccagcgattaggcgggctaccttcttcgagagctgcctagcagcttgtgggaaaccatgagtgctt
ggactctggggggagtatggtcgcaaggctgaaacttaaaggaattgacggaagggcacaccatggagtggagcctgcgg
cttaatttgactcaacacgggaaagcttaccaagctcagatatgattaggattgacagactaaaagatctttcatgatctcataagt
ggtggtgcatggtcgttcttagttggtggagtgatttgtcaggtcaattccggtaacggacgagacctcgacctgctaactagtg
ggattcattctttcgattgacgaggcaggtcttgctttgattatagggggcaacttctatagtcgggtaggttttgtagtagtctgg
aggagtggatttcaaaaaattaatcttcctagaggtacttctggctctaagccagaggaagtccgaggcaataacaggtctgtg
atgcccttagatatcttgggccgcacgcgtgctacaatgtaggcgctaatgagtttttacatccatctccgcaaggagtatggtaa
tcttggaatcacctgcgtgattgggcttgaccactgtaactgtggtcatcaacgaggaattccttgtatgcgcgagtcactatctcg
cgcagaatctgtccctgccctttgtacacaccgcccgtcgctcctaccgatcgaacgatcaggtaaagtggacagagggtacttg
gaagtccattgaacgccg-ttagaggaag
>HQ141518.1_Cavenderia_pseudoaureostipes_TH39A
catgcatgtctaagtataagctcttgtacggctagactgcagacggctcattacaacggttgtatcttccagggcatgtcgcaaga
ccttctggataaccgcagtaaatcggggctaatacatacaaacggagggatggagagggcaaccttgaagtttctgcgatgga
caattagttattcgaccaaccccgcaagggaatggttggaaccggttcatattgctaatcgactctagcttgctagtagtctgataa
gctatagacaaccgccctatcaacttgatggtaaggttttggcttaccatggttgtaacgggtaacggggaatcagggttcgatt
ccggagaggacgcctgagaaacggcgtccacatctacgggtggcagcaggcgcgtaaattgcccaatctcaacagagagg
aggcggtgacaataaatcccgatggctttgggggcaaccccaggctaatcagaataagtacacattaaatcccttaaccaatat
aattggagggcaagtctggtgccagcagccgcggtaattccagctccaatagcatatactaatgttgttgcagttcgctcgtagc
ttaattttgagctatatcgcggctcaacactgtaggtcatggctagcaatagttgcataatcgactgtgcataaaccttgatgctca
aggtaggcctttatagggtagatacacagtgcatggcattgtggaacaaggcatctcgcggcttagttggtgggccgcgggg
gcaatgattaatagggaggagcgggggccttcatattgcagggcgagaggtgaaattcgttgacccttgcaagatgtcctaca
gcgaaagcattggccaagtgcctccccccttagtcaagaacgaaagtttggggatcaaagacgatcagataccgtcgtagtcca
aaccataaactatgtcgaccagcgattaggcgggctaccttcttcgagagctgcctagcagcttgtgggaaaccatgagtgctt
ggactctggggggagtatggtcgcaaggctgaaacttaaaggaattgacggaagggcacaccatggagtggagcctgcgg
cttaatttgactcaacacgggaaagcttaccaagctcagatatgattaggattgacagactaaaagatctttcatgatctcataagt
ggtggtgcatggtcgttcttagttggtggagtgatttgtcaggtcaattccggtaacggacgagacctcgacctgctaactagtg
ggattcattctttcgattgacgaggcaggtcttgctttgattacagggggcaacttctgtagtcgggtacggtttgtagtagtctgg
aggagtgggtttcaaaaaattaatcttcctagaggtacttctggctctaagccagaggaagtccgaggcaataacaggtctgtg
atgcccttagatatcttgggccgcacgcgtgctacaatgtaggcgctaatgagtttttacatccatctccgcaaggagtatggtaa
tcttggaatcacctgcgtgattgggcttgaccactgtaactgtggtcatcaacgaggaattccttgtatgcgcgagtcactatctcg
cgcagaatctgtccctgccctttgtacacaccgcccgtcgctcctaccgatcgaacgatcaggtaaagtggacagagggtacttg
gaagtccattgaacgccg-ttagaggaag
>HQ141523.1_Cavenderia_bhumiboliana_THC11X
catgcatgtctaagtataagctcttgtacggctagactgcagacggctcattacaacggttgtatcttccagggcatgtcgcaaga
ccttctggataaccgcagtaaatcggggctaatacatacaaacggagggatggagagggcaaccttgaagtttctgcgatgga
caattagttattcgaccaaccccgcaagggaatggttggaaccggttcatattgctaatcgactctagcttgctagtagtctgataa
gctatagacaaccgccctatcaacttgatggtaaggttttggcttaccatggttgtaacgggtaacggggaatcagggttcgatt
ccggagaggacgcctgagaaacggcgtccacatctacgggtggcagcaggcgcgtaaattgcccaatctcaacagagagg
aggcggtgacaataaatcccgatggctttgggggcaaccccaggctaatcagaataagtacacattaaatcccttaaccaatat
aattggagggcaagtctggtgccagcagccgcggtaattccagctccaatagcatatactaatgttgttgcagttcgctcgtagc
ttaattttgagctatatcgcggctcaacactgtaggtcatggctagcaatagttgcataatcgactgtgcataaaccttgatgctca
aggtaggcctttatagggtagatacacagtgcatggcattgtggaacaaggcatctcgcggcttagttggtgggccgcgggg
gcaatgattaatagggaggagcgggggccttcatattgcagggcgagaggtgaaattcgttgacccttgcaagatgtcctaca
gcgaaagcattggccaagtgcctccccattagtcaagaacgaaagtttggggatcaaagacgatcagataccgtcgtagtcca

aaccataaactatgtcgaccagcgattaggcgggctaccttcttcgagagctgcctagcagcttgtgggaaaccatgagtgctt
ggactctgggggagtatggtcgcaaggctgaaacttaaaggaattgacggaagggcacaccatggagtggagcctgcgg
cttaatttgactcaacacgggaaagcttaccaagctcagatatgattaggattgacagactaaaagatctttcatgatctcataagt
ggtggtgcatggtcgttcttagttggtggagtgatttgtcaggtcaattccggtaacggacgagacctcgacctgctaactagtg
ggattcattctttcgattgacgaggcaggtcttgctttgattatagggggcaacttctatagtcgggtacggtttgtagtagtctgg
aggagtggatttcaaaaaattaatcttcctagaggtacttctggctctaagccagaggaagtccgaggcaataacaggtctgtg
atgcccttagatatcttgggccgcacgcgtgctacaatgtaggcgctaatgagtttttacatccatctccgcaaggagtatggtaa
tcttggaatcacctgcgtgattgggcttgaccactgtaactgtggtcatcaacgaggaattccttgtatgcgcgagtcactatctcg
cgcagaatctgtccctgccctttgtacacaccgcccgtcgctcctaccgatcgaacgatcaggtaaagtggacagagggttcttg
gaagtccattgaacgccgtttagaggaag
>AM168087.1_Cavenderia_fasciculata_SH3
catgcatgtctaagtataagctcttgtacggctagactgcagacggctcattacaacggttgtatcttacagggcatgtcgcaaga
ccttctggataaccgcagtaaatcggggctaatacatataaacggagggatggagagggtaaccttgaagtttctgcgatgga
catttagctattcgaccaaccccgcaagggaatggttggaaccggttcatattgctaatcgactctagcttgctagtagtctgataa
gctatagacaaccgccctatcaacttgatggtaaggttttggcttaccatggttgtaacgggtaacggggaatcagggttcgatt
ccggagaggacgcctgagaaacggcgtccacatctacgggtggcagcaggcgcgtaaattgcccaatctcaacagagagg
aggcggtgacaataaatcccgatggctttgggggcaaccccaggctaatcagaataagtacacattaaatcccttaaccaatat
aattggagggcaagtctggtgccagcagccgcggtaattccagctccaatagcatatactaatgttgttgcagttcgctcgtagc
ttaattttgagctatatcgcggctcaacactgtaggtcatggctagcaatagttgcataatcgactgtgcataaaccttgatgctca
aggtaggcctttatagggtagatacacagtgcatggcattgtggaacaaggcatctcgcggcttagttggtgggccgcgggg
gcaatgattaatagggaggagcgggggccttcatattgcagggcgagaggtgaaattcgttgacccttgcaagatgtcctaca
gcgaaagcattggccaagtgcctccccattagtcaagaacgaaagtttggggatcaaagacgatcagataccgtcgtagtcca
aaccataaactatgtcgaccagcgattaggcgggctaccttcttcgagagctgcctagcagcttgtgggaaaccatgagtgctt
ggactctgggggagtatggtcgcaaggctgaaacttaaaggaattgacggaagggcacaccatggagtggagcctgcgg
cttaatttgactcaacacgggaaagcttaccaagctcagatatgattaggattgacagactaaaagatctttcatgatctcataagt
ggtggtgcatggtcgttcttagttggtggagtgatttgtcaggtcaattccggtaacggacgagacctcgacctgctaactagtg
ggattcattctttcgattgacgaggcaggtattgctttgattatagggggcaacttttatagtcgggtagtgcttgtattagtctggg
agagtgggtttcaaataattaatcttcctagaggtacttctggctctaagccagaggaagtccgaggcaataacaggtctgtgat
gcccttagatatcttgggccgcacgcgtgctacaatgtaggcgctaatgagtttttacatccatctccgcaaggagtatggtaatct
tggaatcccctgcgtgattgggcttgaccactgtaactgtggtcatcaacgaggaattccttgtatgcgcgagtcactatctcgcg
cagaatctgtccctgccctttgtacacaccgcccgtcgctcctaccgatcgaacgatcaggtaaagtggacagagggttgttgga
agtccattgaacgccgtttagaggaag
>AM168093.1_Cavenderia_delicata_TNS_C_226
catgcatgtctaagtataagctcttgtacggctagactgcagacggctcattacaacggttgtatcttacagggcatgtcgcaaga
ccttctggataaccgcagtaaatcggggctaatacatacaaacggagggatggagagggtaaccttgaagtttctgcgatgga
catttagctattcgaccaaccccgcaagggaatggttggaaccggttcatattgctaatcgactctagcttgctagtagtctgataa
gctatagacaaccgccctatcaacttgatggtaaggttttggcttaccatggttgtaacgggtaacggggaatcagggttcgatt
ccggagaggacgcctgagaaacggcgtccacatctacgggtggcagcaggcgcgtaaattgcccaatctcaacagagagg
aggcggtgacaataaatcccgatggctttgggggcaaccccaggctaatcagaataagtacacattaaatcccttaaccaatat
aattggagggcaagtctggtgccagcagccgcggtaattccagctccaatagcatatactaatgttgttgcagttcgctcgtagc
ttaattttgagctatatcgcggctcaacactgtaggtcatggctagcaatagttgcataatcgactgtgcataaaccttgatgctca
aggtaggcctttatagggtagatacacagtgcatggcattgtggaacaaggcatctcgcggcttagttggtgggccgcgggg
gcaatgattaatagggaggagcgggggccttcatattgcagggcgagaggtgaaattcgttgacccttgcaagatgtcctaca
gcgaaagcattggccaagtgcctccccattagttaagaacgaaagtttggggatcaaagacgatcagataccgtcgtagtcca

aaccataaactatgtcgaccagcgattaggcgggctaccttcttcgagagctgcctagcagcttgtgggaaaccatgagtgctt
ggactctgggggagtatggtcgcaaggctgaaacttaaaggaattgacggaagggcacaccatggagtggagcctgcgg
cttaatttgactcaacacgggaaagcttaccaagctcagatatgattaggattgacagactaaaagatctttcatgatctcataagt
ggtggtgcatggtcgttcttagttggtggagtgatttgtcaggtcaattccggtaacggacgagacctcgacctgctaactagtg
ggattcattctttcgattgacgaggcaggtattgctttgattataggggcaacttttatagtcgggtagtgcttgtattagtctggg
agagtgggtttcaaataattaatcttcctagaggtacttctggctctaagccagaggaagtccgaggcaataacaggtctgtgat
gcccttagatatcttgggccgcacgcgtgctacaatgtaggcgctaatgagtttttacatccatctccgcaaggagtatggtaatct
tggaatcccctgcgtgattgggcttgaccactgtaactgtggtcatcaacgaggaattccttgtatgcgcgagtcactatctcgcg
cagaatctgtccctgccctttgtacacaccgcccgtcgctcctaccgatcgaacgatcaggtaaagtggacagagagtttttgga
agtccattgaacgccgtttagaggaag
>AM168086.1_Cavenderia_fasciculata_SmokOW9A
catgcatgtctaagtataagctcttgtacggctagactgcagacggctcattacaacggttgtatcttacagggcatgtcgcaaga
ccttctggataaccgcagtaaatcggggctaatacatacaaacggagggatggagagggcaaccttgaagtttctgcgatgga
catttagctattcgaccaaccccgcaagggaatggttggaaccggttcatattgctaatcgactctagcttgctagtagtctgataa
gctatagacaaccgccctatcaacttgatggtaaggttttggcttaccatggttgtaacgggtaacggggaatcagggttcgatt
ccggagaggacgcctgagaaacggcgtccacatctacgggtggcagcaggcgcgtaaattgcccaatctcaacagagagg
aggcggtgacaataaatcccgatggctttgggggcaaccccaggctaatcagaataagtacacattaaatcccttaaccaatat
aattggagggcaagtctggtgccagcagccgcggcaattccagctccaatagcatatactaatgttgttgcagttcgctcgtagc
ttaattttgagctatatcgcggctcaacactgtaggtcatggctagcaatagttgcataatcgactgtgcataaaccttgatgctca
aggtaggcctttatagggtagatacacagtgcatggcattgtggaacaaggcatctcgcggcttagttggtgggccgcgggg
gcaatgattaatagggaggagcgggggccttcatattgcagggcgagaggtgaaattcgttgacccttgcaagatgtcctaca
gcgaaagcattggccaagtgcctccccattagtcaagaacgaaagtttggggatcaaagacgatcagataccgtcgtagtcca
aaccataaactatgtcgaccagcgattaggcgggctaccttcttcgagagctgcctagcagcttgtgggaaaccatgagtgctt
ggactctgggggagtatggtcgcaaggctgaaacttaaaggaattgacggaagggcacaccatggagtggagcctgcgg
cttaatttgactcaacacgggaaagcttaccaagctcagatatgattaggattgacagactaaaagatctttcatgatctcataagt
ggtggtgcatggtcgttcttagttggtggagtgatttgtcaggtcaattccggtaacggacgagacctcgacctgctaactagtg
ggattcattctttcgattgacgaggcaggtattgctttgattataggggcaacttttatagtcgggtagtgcttgtattagtctggg
agagtgggtttcaaataattaatcttcctagaggtacttctggctctaagccagaggaagtccgaggcaataacaggtctgtgat
gcccttagatatcttgggccgcacgcgtgctacaatgtaggcgctaatgagtttttacatccatctccgcaaggagtatggtaatct
tggaatcccctgcgtgattgggcttgaccactgtaactgtggtcatcaacgaggaattccttgtatgcgcgagtcactatctcgcg
cagaatctgtccctgccctttgtacacaccgcccgtcgctcctaccgatcgaacgatc-
ggtaaagtggacagagggtttttggaagtccattgaacgccgtttagaggaag
>HQ141516.1_Cavenderia_sp._TAS30A
catgcatgtctaagtataagctcttgtacggctagactgcagacggctcattacaacggttgtatcttacagggcatgtcgcaaga
ccttctggataaccgcagtaaatcggggctaatacatacaaacggagggatggagagggcaaccttgaagtttctgcgatgga
catttagctattcgaccaaccccgcaagggaatggttggaaccggttcatattgctaatcgactctagcttgctagtagtctgataa
gctatagacaaccgccctatcaacttgatggtaaggttttggcttaccatggttgtaacgggtaacggggaatcagggttcgatt
ccggagaggacgcctgagaaacggcgtccacatctacgggtggcagcaggcgcgtaaattgcccaatctcaacagagagg
aggcggtgacaataaatcccgatggctttgggggcaaccccaggctaatcagaataagtacacattaaatcccttaaccaatat
aattggagggcaagtctggtgccagcagccgcggtaattccagctccaatagcatatactaatgttgttgcagttcgctcgtagc
ttaattttgagctatatcgcggctcaacactgtaggtcatggctagcaatagttgcataatcgactgtgcataaaccttgatgctca
aggtaggcctttatagggtagatacacagtgcatggcattgtggaacaaggcatctcgcggcttagttggtgggccgcgggg
gcaatgattaatagggaggagcgggggccttcatattgcagggcgagaggtgaaattcgttgacccttgcaagatgtcctaca
gcgaaagcattggccaagtgcctccccattagtcaagaacgaaagtttggggatcaaagacgatcagataccgtcgtagtcca

aaccataaactatgtcgaccagcgattaggcgggctaccttcttcgagagctgcctagcagcttgtgggaaaccatgagtgctt
ggactctgggggagtatggtcgcaaggctgaaacttaaaggaattgacggaagggcacaccatggagtggagcctgcgg
cttaatttgactcaacacgggaaagcttaccaagctcagatatgattaggattgacagactaaaagatctttcatgatctcataagt
ggtggtgcatggtcgttcttagttggtggagtgatttgtcaggtcaattccggtaacggacgagacctcgacctgctaactagtg
ggattcattctttcgattgacgaggcaggtattgctttgattatagggggcaacttttatagtcgggtagtgcttgtattagtctggg
agagtgggtttcaaataattaatcttcctagaggtacttctggctctaagccagaggaagtccgaggcaataacaggtctgtgat
gcccttagatatcttgggccgcacgcgtgctacaatgtaggcgctaatgagtttttacatccatctccgcaaggagtatggtaatct
tggaatcccctgcgtgattgggcttgaccactgtaactgtggtcatcaacgaggaattccttgtatgcgcgagtcactatctcgcg
cagaatctgtccctgccctttgtacacaccgcccgtcgctcctaccgatcgaacgatcaggtaaagtggacagagggtttttgga
agtccattgaacgccg-ttagaggaag
>AM168080.1_Cavenderia_antarctica_NZ43B
catgcatgtctaagtataagctcttgtacggctagactgcagacggctcattacaacggttgtatcttacagggcatgtcgcaaga
ccttctggataaccgcagtaaatcggggctaatacatacaaacggagggatggagagggcaaccttgaagtttctgcgatgga
catttagctattcgaccaaccccgcaagggaatggttggaaccggttcatattgctaatcgactctagcttgctagtagtctgataa
gctatagacaaccgccctatcaacttgatggtaaggttttggcttaccatggttgtaacgggtaacggggaatcagggttcgatt
ccggagaggacgcctgagaaacggcgtccacatctacgggtggcagcaggcgcgtaaattgcccaatctcaacagagagg
aggcggtgacaataaatcccgatggctttgggggcaaccccaggctaatcagaataagtacacattaaatcccttaaccaatat
aattggagggcaagtctggtgccagcagccgcggtaattccagctccaatagcatatactaatgttgttgcagttcgctcgtagc
ttaattttgagctatatcgcggctcaacactgtaggtcatggctagcaatagttgcataatcgactgtgcataaaccttgatgctca
aggtaggcctttatagggtagatacacagtgcatggcattgtggaacaaggcatctcgcggcttagttggtgggccgcgggg
gcaatgattaataggaaggagcgggggccttcatattgcagggcgagaggtgaaattcgttgacccttgcaagatgtcctaca
gcgaaagcattggccaagtgcctccccattagtcaagaacgaaagtttggggatcaaagacgatcagataccgtcgtagtcca
aaccataaactatgtcgaccagcgattaggcgggctaccttcttcgagagctgcctagcagcttgtgggaaaccatgagtgctt
ggactctgggggagtatggtcgcaaggctgaaacttaaaggaattgacggaagggcacaccatggagtggagcctgcgg
cttaatttgactcaacacgggaaagcttaccaagctcagatatgattaggattgacagactaaaagatctttcatgatctcataagt
ggtggtgcatggtcgttcttagttggtggagtgatttgtcaggtcaattccggtaacggacgagacctcgacctgctaactagtg
ggattcattctttcgattgacgaggcaggtattgctttgattatagggggcaacttttatagtcgggtagtgcttgtattagtctggg
agagtgggtttcaaataattaatcttcctagaggtacttctggctctaagccagaggaagtccgaggcaataacaggtctgtgat
gcccttagatatcttgggccgcacgcgtgctacaatgtaggcgctaatgagtttttacatccatctccgcaaggagtatggtaatct
tggaatcccctgcgtgattgggcttgaccactgtaactgtggtcatcaacgaggaattccttgtatgcgcgagtcactatctcgcg
cagaatctgtccctgccctttgtacacgccgcccgtcgctcctaccgatcgaacgatcaggtaaagtggacagagggtttttgga
agtccattgaacgccgtttagaggaag
>HQ141519.1_Cavenderia_macrocarpa_MGE2
catgcatgtctaagtataagcccttgtacggctagactgcagacggctcattacaacggttgcatcttccagagcatgtcgcaag
accttctggataaccgcagtaaatcggggctaatacatacaaacggaggggtggaccaggcaactgggaaacttctgcgatg
gacacttagctattcgactgaccctttcggggaatggttggaaccggttcatattgctaatcgattgtggcttgccacaagtctgat
aagctataggcaaccgccctatcaacttgatggtaaggttttggcttaccatggttgtaacgggtaacggggaatcagggttcg
attccggagaggacgcctgagaaacggcgtccacatctacgggtggcagcaggcgcgtaaattgcccaatctcaatagagag
gaggcggtgacaataaatcccaatgaccatgggggcaaccccgggtcaattagaataagtacacattaaataccctaaccaat
ataattggagggcaagtctggtgccagcagccgcggtaattccagctccaatagcgtatactaatgttgttgcagttcgctcgta
gctaaattgatggcaattcttaggcc--
agcaagtaggtagtggcatgagggtgttcgacgatcgactgtgcataaaccttgatgctcaaggtaaacatcttcttgaaagat
acacggtgcatggcattgtggaatatggcgttttgtgtccttgttggtgggacacaagagcaatgattaatagggaggagcgg
gggccttcatattgccgggcgagaggtgaaattcgttgaccctggcaagatgtcctacagcgaaagcattggccaagtgcctct

ccattagtcaagaacgaaagtttggggatcaaagacgatcagataccgtcgtagtccaaaccataaactatgtcgaccagcgat
taggttcgccaccttcttcgagagcaacctggcagcttgtgggaaaccatgagttcttggactctggggggagtatggtcgcaa
ggctgaaacttaaaggaattgacggaagggcacaccatggagtggagcctgcggcttaatttgactcaacacgggaaagctt
accaagctcagatatgataaggattgacagaccaaaagatctttcatgatctcataagtggtggtgcatggccgttcttagttggt
ggagtgatttgtcaggtcaattccggtaacggacgagacctcgacctgctaactagtggcagttgtcttttcgatcggcgaggc
gtcgtgggcatggaacttctaggggcaactctatggctgggaagtgtatgtagtagtctggagaggcagctgtccccaatata
atcttcctagaggtacttccagctctaagttggaggaagttcgaggcaataacaggtctgtgatgcccttagatatcttgggccgc
acgcgtgctacaatgtaggcgctaataagaatttatccccggcaccgccaagtgtccggtaatc-
cacaatcacctgcgtgattgggattggtcactgtaactgtgaccatcaacgaggaattccttgtatgcgcgggtcactatcccgc
gcagaatctgtccctgccctttgtacacaccgcccgtcgctcctaccgatcgaacggtaaggtaaagtggacagagaggagct
gaaagtccattgaacgtcg-ttagaggaag
>AM168091.1_Cavenderia_parvispora_OS126
catgcatgtctaagtataagccctagtacggctagactgcagacggctcattacaacggttgcagcttacagagcatgtcgcaa
gaccttctggataaccgcagtaaatcggggctaatacatacaaacggaggggcggagcgggcaaccgcgaagcttctgcga
tggacacttagctattcgactgacccctcacgggaatggttggaaccggttcatattgctaatcgactgtggcttgcctcaagtct
gataagctataggcaaccgccctatcaacttgatggtaaggtattggcttaccatggttgtaacgggtaacggggaatcagggt
tcgattccggagaggacgcctgagaaacggcgtccacatctacgggtggcagcaggcgcgtaaattgcccaatctcaataga
gaggaggcggtgacaataaatcccgatggccatgggggcaaccccgggccaatcagaataagtacacattaaataccctaac
caatataattggagggcaagtctggtgccagcagccgcggtaattccagctccaatagcgtatactaatgttgttgcagttcgctc
gtagctgaattgatggcaattcagcggct-
aagcaggtaggtagtcgtgtgtaggcgtcaggcgatcgactgtgcataaaccttgatgctcaaggtaaagactctagtcaaag
atacgcagtgcatggcattgtggaacaaggcgatccgtagcttggttggtgagctacgggtgcaatgattaatagggaggag
cgggggccttcatattgccgggcgagaggtgaaattcgttgaccctggcaagatgtcctacagcgaaagcattggccaagtgc
ctctccattagtcaagaacgaaagtttggggatcaaagacgatcagataccgtcgtagtccaaaccataaactatgtcgaccagc
gattaggttcgccaccttcttcgagagcaacctggcagcttgtgggaaaccatgagttcttggactctggggggagtatggtcg
caaggctgaaacttaaaggaattgacggaagggcacaccatggagtggagcctgcggcttaatttgactcaacacgggaaa
gcttaccaagctcagatatgataaggattgacagaccaaaagatctttcatgatctcataagtggtggtgcatggccgttcttagtt
ggtggagtgatttgtcaggtcaattccggtaacggacgagacctcgacctgctaactagtagcagttgtcttttcgctcggcgag
gcgttttgggcgtggaactttgaggggcaactctatctttgggaaacgagcgtagtagtctggagaggcagctgttcccaaaat
aatcttcctagaggtacttccagctctaagttggaagaagtccgaggcaataacaggtctgtgatgcccttaaatatcttgggccg
cacgcgtgctacaatgtaggcgctaatgagtcaatat--ccggcaccgcaaggtgtctggtaatc-
cccaatcccctgcgtgattgggattggtcactgtaactgtgaccatcaacgaggaattccttgtatgcgcgggtcactatcccgcg
cagaatctgtccctgccctttgtacacaccgcccgtcgctcctaccgatcgaacggtaaggtaaagtggacggaggatggctgg
aagtccattgaacgtcgtttagaggaag
>HQ141521.1_Cavenderia_amphispora_BM9A
catgcatgtctaagtataagccctagtacggctagactgcagacggctcattacaacggttgcagcttacagagcatgtcgcaa
gaccttctggataaccgcagtaaatcggggctaatacatacaaacggaggggcggagcgggcaaccgcgaagcttctgcga
tggacacttagctattcgactgacccctcacgggaatggttggaaccggttcatattgctaatcgactgtggcttgcc-
caagtctgataagctataggcaaccgccctatcaacttgatggtaaggtattggcttaccatggttgtaacgggtaacggggaat
cagggttcgattccggagaggacgcctgagaaacggcgtccacatctacgggtggcagcaggcgcgtaaattgcccaatctc
aatagagaggaggcggtgacaataaatcccgatggccatgggggcaaccccgggccaatcagaataagtacacattaaata
ccctaaccaatataattggagggcaagtctggtgccagcagccgcggtaattccagctccaatagcgtatactaatgttgttgca
gttcgctcgtagctgaattgatggcaattcagcggct-
aagcaggtaggtagtcgtgtgtaggcgtcaggcgatcgactgtgcataaaccttgatgctcaaggtaaagactctagtcaaag

atacgcagtgcatggcattgtggaacaaggcgatccgtagcttggttggtgagctacgggtgcaatgattaatagggaggag
cgggggccttcatattgccgggcgagaggtgaaattcgttgaccctggcaagatgtcctacagcgaaagcattggccaagtgc
ctctccattagtcaagaacgaaagtttggggatcaaagacgatcagataccgtcgtagtccaaaccataaactatgtcgaccagc
gattaggttcgccaccttcttcgagagcaacctggcagcttgtgggaaaccatgagttcttggactctgggggagtatggtcg
caaggctgaaacttaaaggaattgacggaagggcacaccatggagtggagcctgcggcttaatttgactcaacacgggaaa
gcttaccaagctcagatatgataaggattgacagaccaaaagatctttcatgatctcataagtggtggtgcatggccgttcttagtt
ggtggagtgatttgtcaggtcaattccggtaacggacgagacctcgacctgctaactagtggcagttgtcttttcgctcggcgag
gcgttttgggcgtggaactttgaggggcaactctatctttgggaagcgagc-
tagtagtctggagaggcagctgtccccaaaataatcttcctagaggtacttccagctctaagttggaggaagtccgaggcaata
acaggtctgtgatgcccttagatatcttgggccgcacgcgtgctacaatgtaggcgctaatgagtcaatat-
cccggcaccgccaagtgtctggtaatc-
cccaatcccctgcgtgattgggattggtcactgtaactgtgaccatcaacgaggaattccttgtatgcgcgggtcactatcccgcg
cagaatctgtccctgccctttgtacacaccgcccgtcgctcctaccgatcgaacggtaaggtaaagtggacggaggatggctgg
aagtccattgaacgtcg-ttagaggaag
>AM168090.1_Cavenderia_microspora_TNS_C_38
catgcatgtctaagtataagccctagtacggctagactgcagacggctcattacaacggttgcagcttacagagcatgtcgcaa
gaccttctggataaccgcagtaaatcggggctaatacatacaaacggaggggcggagcgggcaaccgcgaagcttctgcga
tggacacttagctattcgactgacccctcacgggaatggttggaaccggttcatattgctaatcgactgtggcttgcctcaagtct
gataagctataggcaaccgccctatcaacttgatggtaaggtattggcttaccatggttgtaacgggtaacggggaatcagggt
tcgattccggagaggacgcctgagaaacggcgtccacatctacgggtggcagcaggcgcgtaaattgcccaatctcaataga
gaggaggcggtgacaataaatcccgatggccatgggggcaaccccgggccaatcagaataagtacacattaaataccctaac
caatataattggagggcaagtctggtgccagcagccgcggtaattccagctccaatagcgtatactaatgttgttgcagttcgctc
gtagctgaattgatggcaattcagcggct-aagcaggtaggtagtcg-gtgtag-
cgtcaggcgatcgactgtgcataaaccttgatgctcaaggtaaagattctagtcaaagatacgcagtgcatggcattgtggaac
aaggcgatccgtagcttggttggtgagctacgggtgcaatgattaatagggaggagcgggggccttcatattgccgggcgag
aggtgaaattcgttgaccctggcaagatgtcctacagcgaaagcattggccaagtgcctctccattagtcaagaacgaaagtttg
gggatcaaagacgatcagataccgtcgtagtccaaaccataaactatgtcgaccagcgattaggttcgccaccttcttcgagag
caacctggcagcttgtgggaaaccatgagttcttggactctgggggagtatggtcgcaaggctgaaacttaaaggaattgac
ggaagggcacaccatggagtggagcctgcggcttaatttgactcaacacgggaaagcttaccaagctcagatatgataagga
ttgacagaccaaaagatctttcatgatctcataagtggtggtgcatggccgttcttagttggtggagtgatttgtcaggtcaattcc
ggtaacggacgagacctcgacctgctaactagtggcagttgtcttttcgctcggcgaggcgttttgggcgtggaactttgaggg
gcaactctatctttgggaagcgagcgtagtagtctggagaggcagctgtccccaaaataatcttcctagaggtacttccagctct
aagttggaggaagtccgaggcaataacaggtctgtgatgcccttagatatcttgggccgcacgcgtgctacaatgtaggcgct
aatgagtcaatat--ccggcaccgcaaggtgtctggtaatc-
cccaatcccctgcgtgattgggattggtcactgtaactgtgaccatcaacgaggaattccttgtatgcgcgggtcactatcccgcg
cagaatctgtccctgccctttgtacacaccgcccgtcgctcctaccgatcgaacggtaaggtaaagtggacggaggatggctgg
aagtccattgaacgtcgtttagaggaag
>HQ141520.1_Cavenderia_boomerangispora_K26B
catgcatgtctaagtataagcccttgtacggctagactgcagacggctcattacaacggttgtagcttacagagcatgc--
gcaagccttctggataaccgcagtaaatcggggctaatacatacaaacggaggggtggagcgggcaaccgcaaagcttctg
cgatggacacttagctattcgactaaccccta--cgggtcaggttggaaccggttcatattgccaatcgactatggcttgcca-
tagtctgataagctatagacaaccgccctatcaacttgatggtaaggtattggcttaccatggttgtaacgggtaacggggaatc
agggttcgattccggagaggacgcctgagaaacggcgtccacatctacgggtggcagcaggcgcgtaaattgcccaatctca
atagagaggaggcggcgacaataaatcccgatggctatgggggcaaccccgggccaatcagaataagtacacaataaatac

cctaaccaatataattggagggcaagtctggtgccagcagccgcggtaattccagctccaatagcatatactaacgttgttgcag
ttcgctcgtagctgaattgatggcaattcagcggcc-
aagcaagtaggtagtggagtaggggcttcaggcgatcgactgtgcataaaccttgatgctcaaggtaaagactctagtcaaag
atacgcagtgcatggcattgtggaacaaggcgatccgtagcttagttagtgggctgcgggtgcaatgattaatagggaggag
cgggggccttcatattgccgggcgagaggtgaaattcgttgaccctggcaagatgtcctacagcgaaagcattggccaagtgc
ctctccattagtcaagaacgaaagtttggggatcaaagacgatcagataccgtcgtagtccaaaccataaacgatgtcgaccag
cgattaggttcgccaccttcttcgagagcaacctggcagcttgtgggaaaccatgagttcttggactctgggggagtatggtc
gcaaggctgaaacttaaaggaattgacggaagggcacaccatggagtggagcctgcggcttaatttgactcaacacgggaa
agcttaccaagctcagatatgataaggattgacagaccaatagatctttcatgatct------------------------------
----

>AM168085.1_Cavenderia_exigua_TNS_C_199
catgcatgtctaagtataagcccttgtacggctagactgcagacggctcattacaacggttgtagcttacagagcatgc--
gcaagccttctggataaccgcagtaaatcggggctaatacatacaaacggaggggtggagcgggcaaccgcaaagcttctg
cgatggacacttagctattcgactaacccttt--cgggtcaggttggaaccggttcatattgccaatcgactatggcttgcca-
tagtctgataagctatagacaaccgccctatcaacttgatggtaaggtattggcttaccatggttgtaacgggtaacggggaatc
agggttcgattccggagaggacgcctgagaaacggcgtccacatctacgggtggcagcaggcgcgtaaattgcccaatctca
atagagaggaggcggcgacaataaatcccgatggctatgggggcaaccccgggccaatcagaataagtacacaataaatac
cctaaccaatataattggagggcaggtctggtgccagcagccgcggtaattccagctccaatagcatatactaacgttgttgcag
ttcgctcgtagctgaattgatggcaattcagcggcc-
aagcaagtaggtagtgatgtgagggcgtcaggcgatcgactgtgcataaaccttgatgcttaaggtaaagattctagtcaaag
atacgcagtgcatggcattgtggaacaaggcgatccgtagcttagttagtgggctgcgggtgcaatgattaatagggaggag
cgggggccttcatattgccgggcgagaggtgaaattcgttgaccctggcaagatgtcctacagcgaaagcattggccaagtgc
ctctccattagtcaagaacgaaagtttggggatcaaagacgatcagataccgtcgtagtccagaccataaacgatgtcgaccag
cgattaggttcgccaccttcttcgagagcaacctggcagcttgtgggaaaccatgagttcttggactctgggggagtatggtc
gcaaggctgaaacttaaaggaattgacggaagggcacaccatggagtggagcctgcggcttaatttgactcaacacgggaa
agcttaccaagctcagatatgataaggattgacagaccaatagatctttcatgatctcataagtggtggtgcatggccgttcttagt
tggtggagtgatttgtcaggtcaattccggtaacggacgagacctcgacctgctaactagtggcagttgtcttttcgctcggcga
ggcgtcattggcatacgactttgaggggcaactctatcttggaagtgtgagcgtagtagtctggagaggcagctgtcccctaatt
aatcttcctagaggtacttccagctctaagctggaggaagtccgaggcaataacaggtctgtgatgcccttagatatcttgggcc
gcacgcgtgctacaatgtaggcgctaatatgatattac--ccggcaccgtcaaggtgtcggtaatc-
tccaatcacctgcgtgattgggcttggtcactgtaactgtgaccattaacgaggaattccttgtatacgcgagtcactatctcgcat
agaatctgtccctgccctttgtacacaccgcccgtcgctcctaccgatcgaacggtaaggtaaagtggacagacggtagctgga
agtccattgaacttcgtttagaggaag
>AM168030.1_Dictyostelium_brefeldianum_TNS_C_115
catgcatgtctaagtataaattcttgtacgatgaaactgcagacggctcattacaacagtgataaactgctagacttgttttta-
acctttggataaccgcagtaaatcggggctaatacatagaagcgatggg------
cgactggtaacggaagctcagcgatta----
ttagcataactaccaataccttcgggtcttgggtgaaaccgaataatattgcagatcgaa--------

gatttatcttcgacaagtaatgtgtcactgccctatcaacttgatggtacggtattggcctaccatggttgtaacgggtaacgggg
aattagggttcgattccggagagggagcctgagaaatggctaccacttctacggaaggcagcaggcgcgcaaattactcaatc
ccaata-cggggaagtagtgacaataaatatcaatacctat---
ccttaatggagggcaattgaaatgaacacaaattaaaactcttaattaacacaattggagggcaagtctggtgccagcagccgc
ggtaattccagctccaatagcatatactaaagttgttgcagttagctcgtagttgaatattgggtcaagctaatgctcaattgttcag
cttgttcaactttgcgtttggacatttcactgtgagaaaattgtggtgtttaaagcaggcg--
tctcgcctgatcttttgcagcatggtatgatgaaacatgacattttgcgctattggtt-
tgcgtctaaagtgtaatgattaatagggatggatgggggtgttcatattggtgggcgagaggtgaaattcgttgaccctatcaa
gatgaacttctgcgaaagcattcaccaaatacttctccattaatcaagaacgaaagtttggggatcgaagacgatcagataccgt
cgtagtccaaactataaactatgtcgaccagggatcggttaa---
aattttttaaaatttaatcggcaccttgtgagaaatcacgagtgtttagattccgggggagtatggtcgcaagtctgaaacttaa
aggaattgacggaagggcacacaatggagtggagcctgcggcttaatttgactcaactcgggaaaacttaccaagctaagat
atagttaggattgacagactaaaagatctttcatgattctataagtggtggtgcatggtcgttcttagttggtggagcgatttgtct
ggtcaattccgataacggacgagacctcgacctgctaactagtagtatttattagtcaaatgggcgatagcttttctggatttaga
gcgatcggtcg-tttctggttcaaggagtgtg-----
tagtctgacttgataggtacgaattaaaaaaacttcttagagggactacctgcctcaagcaggcggaagtccgaggcaataaca
ggtctgtgatgcccttagataccttgggccgcacgcgcgctacaatgtataaaacaaaaaggt------
tcctggtccggaaggattgggtaatcaattgaattttctacgtaactgggattgatctttgtaattatgatcataaacgaggaattcc
ttgtaagcgtaggtcattaccctatgctgaatatgtccctgccctttgtacacaccgcccgtcgctcctaccgatcgaatgatacggt
aaagttaacggatataaattaaaagttatttaaacattgtttagaggaag
>HQ141482.1_Dictyostelium_mucoroides_sweden_20
catgcatgtctaagtataaattcttgtacgatgaaactgcagacggctcattacaacagtgataaactgctagacttgttttta-
accttttggataaccgcagtaaatcggggctaatacatagaagcgatggg------
cgactggtaacggaagctcagcgatta----
ttagcataactaccaataccttcgggtcttgggtgaaaccgaataatattgcagatcgaa--------
gatttatcttcgacaagtaatgtgtcactgccctatcaacttgatggtacggtattggcctaccatggttgtaacgggtaacgggg
aattagggttcgattccggagagggagcctgagaaatggctaccacttctacggaaggcagcaggcgcgcaaattactcaatc
ccaata-cggggaagtagtgacaataaatatcaatacctat---
ccttaatggagggcaattgaaatgaacacaaattaaaactcttaattaacacaattggagggcaagtctggtgccagcagccgc
ggtaattccagctccaatagcatatactaaagttgttgcagttagctcgtagttgaatattgggtcaagctaatgctcaattgttcag
cttgttcaactttgcgtttggacatttcactgtgagaaaattgtggtgtttaaagcaggcg--
tctcgcctgatcttttgcagcatggtatgatgaaacatgacattttgcgctattggtt-
tgcgtctaaagtgtaatgattaatagggatggatgggggtgttcatattggtgggcgagaggtgaaattcgttgaccctatcaa
gatgaacttctgcgaaagcattcaccaaatacttctccattaatcaagaacgaaagtttggggatcgaagacgatcagataccgt
cgtagtccaaactataaactatgtcgaccagggatcggttaa---
aattttttaaaatttaatcggcaccttgtgagaaatcacgagtgtttagattccgggggagtatggtcgcaagtctgaaacttaa
aggaattgacggaagggcacacaatggagtggagcctgcggcttaatttgactcaactcgggaaaacttaccaagctaagat
atagttaggattgacagactaaaagatctttcatgattctataagtggtggtgcatggtcgttcttagttggtggagcgatttgtct
ggtcaattccgataacggacgagacctcgacctgctaactagtagtatttattagtcaaatgggcgatagcttttctggatttaga
gcgatcggtcg-tttctggttcaaggagtgtg-----
tagtctgacttgataggtacgaattaaaaaaacttcttagagggactacctgcctcaagcaggcggaagtccgaggcaataaca
ggtctgtgatgcccttagataccttgggccgcacgcgcgctacaatgtataaaacaaaaaggt------
tcctggtccggaaggattgggtaatcaattgaattttctacgtaactgggattgatctttgtaattatgatcataaacgaggaattcc
ttgtaagcgtaggtcattaccctatgctgaatatgtccctgccctttgtacacaccgcccgtcgctcctaccgatcgaatgatacggt

aaagttaacggatataaattaaaagttatttaaacattgtttagaggaag
>AM168049.1_Dictyostelium_macrocephalum_B33
catgcatgtctaagtataaattcttgtacgatgaaactgcagacggctcattacaacagtgataaactaatagacttgttttta-
accttttggataaccgcagtaaatcggggctaatacatacaagcgatggg------
tgactggcaacggaagctcagcgatta----
ttagcattactaccaataccttcgggtcttgggtgaaaccgaataatattgcagatcgag--------
gatttatcttcgacaagtactgtgtcactgccctatcaacttgatggtacggtattggcctaccatggttgtaacgggtaacgggg
aattagggttcgattccggagagggagcctgagaaatggctaccacttctacggaaggcagcaggcgcgcaaattactcaatc
ccaata-cggggaagtagtgacaataaatatcaatacctat---
ccttaacggagggcaattgaaatgaacacaaattaaaactcttaattaacacaattggagggcaagtctggtgccagcagccgc
ggtaattccagctccaatagcatatactaaagttgttgcagttagctcgtagttgaatactgggctaagttacaactcaatagctct
gcttctgcatctttgtgtttgggcatttcactgtgagaaaattgtggtgtttaaagcaggcg--
tctcgcctgatcttttgcagcatggtatgatgaaacatgacattttacgctattggtt-
tgcgtctaaagtgtaatgattaatagggatggatgggggtgttcatattggtgggcgagaggtgaaattcgttgaccctatcaa
gatgaacttctgcgaaagcattcaccaaatacttccccattaatcaagaacgaaagtttggggatcgaagacgatcagataccgt
cgtagtccaaactataaactatgtcgaccagggatcggtcaa---
aattttttaaaatttgatcggcaccttgtgagaaatcatgagtgtttagattccgggggagtatggtcgcaagtctgaaacttaa
aggaattgacggaagggcacacaatggagtggagcctgcggcttaatttgactcaactcgggaaaacttaccaagctaagat
atagtaaggattgacagactaaaagatctttcatgattctataagtggtggtgcatggtcgttcttagttggtggagcgatttgtct
ggtcaattccgataacggacgagacctcgacctgctaactagtagtatttattagccgaatgggcgatagcttctctgggtttgga
atgatcggtca-tctcctattcaaggagtgtg-----tagtctggcttgataggtacgaa-
ttaaaaaacttcttagagggactacctgcctcaagcaggcggaagtccgaggcaataacaggtctgtgatgcccttagatacctt
gggccgcacgcgcgctacaatgtagaaaacaaaaaggc------
tcctggtccggaaggattgggtaatcatttgaattttctacgtaactgggattgatctttgtaattatgatcatcaacgaggaattcct
tgtaagcgcaggtcattaccctgtgctgaatatgtccctgccctttgtacacaccgcccgtcgctcctaccgatcgaatgatacggt
aaagttaacagatataaactaaaagttatttaaacattgtttagaggaag
>AM168050.1_Dictyostelium_medium_TNS_C_205
catgcatgtctaagtataaattcttgtacgatgaaactgcagacggctcattacaacagtgataaactaatagacttgttttta-
accttttggataaccgcagtaaatcggggctaatacatagaagcgatggg------tgac-ggcaac-
gaagctcagcgatta----ttagcattactaccaataccttcgggtcttgggtgaaaccgaataatattgcagatcgag------
--
gatttatcttcgacaagtactgtgtcactgccctatcaacttgatggtacggtattggcctaccatggttgtaacgggtaacgggg
aattagggttcgattccggagagggagcctgagaaatggctaccacttctacggaaggcagcaggcgcgcaaattactcaatc
ccaata-cggggaagtagtgacaataaatatcaatacctat---
ccttaatggagggcaattgaaatgaacacaaattaaaactcttaattaacacaattggagggcaagtctggtgccagcagccgc
ggtaattccagctccaatagcatatactaaagttgttgcagttagctcgtagttgaatactgggctaagttacaactcaatagttca
gcttgttcaacctagcgtttggacatttcactgtgagaaaattgtggtgtttaaagcaggcg--
tctcgcctgatcttttgcagcatggtatgatggaacatgacattttgcgctattggtt-
tgcgtctaaagtgtaatgattaatagggatggatgggggtgttcatattggtgggcgagaggtgaaattcgttgaccctatcaa
gatgaacttctgcgaaagcattcaccaaatacttccccattaatcaagaacgaaagtttggggatcgaagacgatcagataccgt
cgtagtccaaactataaactatgtcgaccagggatcggttaa---
aattttttaaaatttaatcggcaccttgtgagaaatcacgagtgtttagattccgggggagtatggtcgcaagtctgaaacttaa
aggaattgacggaagggcacacaatggagtggagcctgcggcttaatttgactcaactcgggaaaacttaccaagctaagat
atagtaaggattgacagactaaaagatctttcatgattctataagtggtggtgcatggtcgttcttagttggtggagcgatttgtct

ggtcaattccgataacggacgagacctcgacctgctaactagtagtatttattagtcgaataggcgatagcttttctgggtttgga
atgatcggtca-tctcctattcaaggagtgtg-----tagtctggcttgataggtacgaa-
ttaaaaaacttcttagagggactacctgcctcaagcaggcggaagtccgaggcaataacaggtctgtgatgcccttagatacctt
gggccgcacgcgcgctacaatgtagaaaacaaaaaggc------
tcctggtccggaaggattgggtaatcatttgaattttctacgtaactgggattgatctttgtaattatgatcatcaacgaggaattcct
tgtaagcgcaggtcattaccctgtgctgaatatgtccctgccctttgtacacaccgcccgtcgctcctaccgatcgaatgatacggt
aaagttaacggatataaactaaaagttatttaaacattgtttagaggaag
>MN338956.1_Cavenderia_canoespora_Mad14_3C
----
tataagctcttgtacggctagactgcagacggctcattacaacggttgtatcttacagggcatgtcgcaagaccttctggataacc
gcagtaaatcggggctaatacatataaacggagggatggagagggcaaccttgaagtttctgcgatggacatttagctattcg
accaaccccgcaagggaacggttggaaccggttcatattgctaatcgactctagcttgctagtagtctgatgagctatagacaac
cgccctatcaacttgatggtaaggttttggcttaccatggttgtaacgggtaacggggaatcagggttcgattccggagaggac
gcctgagaaacggcgtccacatctacgggtggcagcaggcgcgtaaattgcccaatctcaacagagaggaggcggtgacaa
taaatcccgatggctttgggggcaaccccaggccaatcagaataagtacacattaaatcccttaaccaatataattggagggca
agtctggtgccagcagccgcggtaattccagctccaatagcatatactaatgttgttgcagttcgctcgtagctgaattttgagcta
tatcgcggctcaacactgtaggtgattattggtagtaatatcataatcgactgtgcataaaccttgatgctcaaggtaggcctttat
agggtagatacacagtgcatggcattgtggaacaaggcatctcgcggcttagttggtgggccgcgggggcaatgattaatag
ggaggagcgggggccttcatattgcagggcgagaggtgaaattcgttgacccttgcaagatgtccgacagcgaaagcattg
gccaagtgcctctccattagtcaagaacgaaagtttggggatcaaagacgatcagataccgtcgtagtccaaaccataaactat
gtcgaccagcgattaggcgggctaccttcttcgagagctgcctagcagcttgtgggaaaccatgagtgcttggactctggggg
gagtatggtcgcaaggctgaaacttaaaggaattgacggaagggcacaccatggagtggagcctgcggcttaatttgactca
acacgggaaagcttaccaagctcagatatgataaggattgacagactaaaagatctttcatgatctcataagtggtggtgcatg
gtcgttcttagttggtggagtgatttgtcaggtcaattccggtaacggacgagacctcgacctgctaactagtgggattcattcttt
cgattgacgaggcgggtattgctttgattatgaggggcaacctttatagtcgggtaatatttgtagtagtctggaggagtgggttt
cagataattaatcttcctagaggtacttctggctctaagccagaggaagtccgaggcaataacaggtctgtgatgcccttagatat
cttgggccgcacgcgtgctacaatgtaggcgctaatgagtcacaatttccagctccgtaaggagtctggtaatcttggaatcacc
tgcgtgattgggcttgactactgtaactgtggtcatcaacgaggaattccttgtatgcgcgagtcactatctcgcgcagaatctgtc
cctgccctttgtacacaccgcccgtcgctcctaccgatcgaacgatcaggtaaagtggacagaggatctttagaagtcca----
----
>MN338955.1_Cavenderia_basinodulosa_Mad5_1A
catgcatgtctaagtaagctcttgctactgttatgactgcagacggctcattatcatatctgtatcttacagggcatgtcgcaagacc
ttctggataaccgcagtaaatcggggctaatacatataaacggagggatggagagggctaccttgaagtttctgcgatggaca
tttagctattcgacctcccccgcaagggaacggttggaaccggttcatattgctaatcgactctagcttgctagttttctgatgagct
atagacaaccgccctatcaacttgatggtaaggttttggcttaccttggttgtatcgggtaacggggaatcagggttcgattccgg
agaggacgcctgacaaacggcgtccacatctacgggtggcagcaggcgcgtaaattgcccaatctcaacagagaggaggc
ggtgacaataaatcccgatggctttgggggcaaccccaggccaatcagaataagtacacattaaatcccttaaccaatataattg
gagggcaagtctggtgccagcagccgcggtaattccagctccaatagcatatactaatgttgttgcagttcgctcgtagctgaat
tttgagctatatcgcggctcaacactgtaggtgattattggtagtaatatcataatcgactgtgcataaaccttgatgctcaaggta
ggcctttatagggtagatacacagtgcatggcattgtggaacaaggcatctcgcggcttagttggtgggccgcgggggcaat
gattaatagggaggagcgggggccttcatattgcagggcgagaggtgaaattcgttgacccttgcaagatgtccgacagcga
aagcattggccaagtgcctctccattagtcaagaacgaaagtttggggatcaaagacgatcagataccgtcgtagtccaaacca
taaactatgtcgaccagcgattaggcgggctaccttcttcgagagctgcctagcagcttgtgggaaaccatgagtgcttggactc
tgggggggagtatggtcgcaaggctgaaacttaaaggaattgacggaagggcacaccatggagtggagcctgcggcttaattt

gactcaacacgggaaagcttaccaagctcagatatgataaggattgacagactaaaagatctttcatgatctcataagtggtggt
gcatggtcgttcttagttggtggagtgatttgtcaggtcaattccggtaacggacgagacctcgacctgctaactagtgggattc
attctttcgattgacgaggcgggtattgctttgattatgaggggcaacctttatagtcgggtaatatttgtagtagtctggaggagt
gggtttcagataattaatcttcctagaggtacttctggctctaagccagaggaagtccgaggcaataacaggtctgtgatgccctt
agatatcttgggccgcacgcgtgctacaatgtaggcgctaatgagtcacaatttccagctccgtaaggagtctggtaatcttgga
atcacctgcgtgattgggcttgactactgtaactgtggtcatcaacgaggaattccttgtatgcgcgagtcactatctcgcgcaga
atctgtccctgccctttgtacacaccgcccgtcgctcctaccgatcgaacgatcaggtaaaatggacattagtcctcccgatgtaat
acggt----------------
>MH745571.1_Cavenderia_aureostabilis_TH10B
----
catgtctaagtataagctcttgtacggctagactgcagacggctcattacaacggttgtatcttccaggacatgtcgcaagatcttc
tggataaccgcagtaaatcggggctaatacatacaaacgaaggggtggagagggcaaccttgaagcttctgcgatggacact
tagctattcgaccaaccccgcaagggagtggttggaaccggttcatattgctaatcgactctggcttgccatgagtc-
gataagctatagacaaccgccctatcaacttgatggtaaggttttggcttaccatggttgtaacgggtaacggggaatcagggtt
cgattccggagaggacgcctgagaaacggcgtccacatctacgggtggcagcaggcgcgtaaattgcccaatctcaacaga
gaggaggcggtgacaataaatcccgatggctttgggggcaaccccaggctaatcagaataagtacacattaaatcccttaacc
aatataattggagggcaagtctggtgccagcagccgcggtaattccagctccaatagcatatactaatgttgttgcagttcgctc
gtagcttaattttgagctattacgcggctcaacactgtaggtcatgcttagcaataagttcataatcgactgtgcataaaccttgatg
ctcaaggtaggcctttatagggtagatacacagtgcatggcattgtggaacaaggcatctcgcggcttagttggtgggccgcg
ggggcaatgattaatagggaggagcgggggccttcatattgcagggcgagaggtgaaattcgttgacccttgcaagatgtcc
gacagcgaaagcattggccaagtgcctccccattagtcaagaacgaaagtttggggatcaaagacgatcagataccgtcgtag
tccaaaccataaactatgtcgaccagcgattaggcgggctaccttcttcgagagctgcctagcagcttgtgggaaaccatgagt
gcttggactctgggggggagtatggtcgcaaggctgaaacttaaaggaattgacggaagggcacaccatggagtggagcctg
cggcttaatttgactcaacacgggaaagcttaccaagctcagatatgataaggattgacagactaaaagatctttcatgatctcat
aagtggtggtgcatggtcgttcttagttggtggagtgatttgtcaggtcaattccggtaacggacgagacctcgacctgctaact
agtgggattcattccttcgctcgacgaggcgggctctgcttttattatgaggg--
caacttcatagtagggtagggttcgtattagtctgggggagtgggtttcaaaaatttaatcttcctagaggtacttctggctctaag
ccagaggaagttcgaggcaataacaggtctgtgatgcccttagatatcttgggccgcacgcgtgctacaatgtaggcgctaatg
agtcaatacatccagctccgtaaggagtctggtaatcttggaatcccctgcgtgattgggcttgactactgtaactgtggtcatca
acgaggaattccttgtatgcgcgagtcactatctcgcgcagaatctgtccctgccctttgtacacaccgcccgtcgctcctaccgat
cgaacgatcaggtaaagtggacagaggcatttggaaagtccattgaacgccgtttagaggaag
>MH745572.1_Cavenderia_protodigitata_TH18BA
cctgcatgtctaagtataagctcttgtacggctagactgcagacggctcattacaacggttgtatcttccagggcatgtcgcaaga
ccttctggataaccgcagtaaatcggggctaatacatacaaacggagggatggagagggcaaccttgaagtttctgcgatgga
caattagttattcgaccaaccccgcaagggaatggttggaaccggttcatattgctaatcgactctagcttgctagtagtctgataa
gctatagacaaccgccctatcaacttgatggtaaggttttggcttaccatggttgtaacgggtaacggggaatcagggttcgatt
ccggagaggacgcctgagaaacggcgtccacatctacgggtggcagcaggcgcgtaaattgcccaatctcaacagagagg
aggcggtgacaataaatcccgatggctttgggggcaaccccaggctaatcagaataagtacacattaaatcccttaaccaatat
aattggagggcaagtctggtgccagcagccgcggtaattccagctccaatagcatatactaatgttgttgcagttcgctcgtagc
ttaattttgagctatatcgcggctcaacactgtaggtcatggctagcaatagttgcataatcgactgtgcataaaccttgatgctca
aggtaggcctttatagggtagatacacagtgcatggcattgtggaacaaggcatctcgcggcttagttggtgggccgcgggg
gcaatgattaatagggaggagcgggggccttcatattgcagggcgagaggtgaaattcgttgacccttgcaagatgtcctaca
gcgaaagcattggccaagtgcctccccattagtcaagaacgaaagtttggggatcaaagacgatcagataccgtcgtagtcca
aaccataaactatgtcgaccagcgattaggcgggctaccttcttcgagagctgcctagcagcttgtgggaaaccatgagtgctt

ggactctgggggagtatggtcgcaaggctgaaacttaaaggaattgacggaagggcacaccatggagtggagcctgcgg
cttaatttgactcaacacgggaaagcttaccaagctcagatatgattaggattgacagactaaaagatctttcatgatctcataagt
ggtggtgcatggtcgttcttagttggtggagtgatttgtcaggtcaattccggtaacggacgagacctcgacctgctaactagtg
ggattcattctttcgattgacgaggcaggtcttgctttgattatagggggcaacttctatagtcgggtacggtttgtagtagtctgg
aggagtggatttcaaaaaattaatcttcctagaggtacttctggctctaagccagaggaagtccgaggcaataacaggtctgtg
atgcccttagatatcttgggccgcacgcgtgctacaatgtaggcgctaatgagtttttacatccatctccgcaaggagtatggtaa
tcttggaatcacctgcgtgattgggcttgaccactgtaactgtggtcatcaacgaggaattccttgtatgcgcgagtcactatctcg
cgcagaatctgtccctgccctttgtacacaccgcccgtcgctcctaccgatcgaacgatcaggtaaagtggacagagggttcttg
gaagtccattgaacgccg-ttagaggaag
>KF662214.1_Cavenderia_aureostipes_var._helvetia_HM592
----
tgtatcttacagggcatgtcgcaagaccttctggataaccgcagtaaatcggggctaatacatacaaacggaggaatggaggg
ggcaaccttgaagtttctgcgatggacatttagctattcgaccaaccccgcaagggaatggttggaaccggttcatattgctaatc
gactctagcttgctagtagtctgataagctatagacaaccgccctatcaacttgatggtaaggttttggcttaccatggttgtaacg
ggtaacggggaatcagggttcgattccggagaggacgcctgagaaacggcgtccacatctacgggtggcagcaggcgcgt
aaattgcccaatctcaacagagaggaggcggtgacaataaatcccgatggctttgggggcaaccccaggctaatcagaataa
gtacacattaaatcccctaaccaatataattggagggcaagtctggtgccagcagccgcggtaattccagctccaatagcatata
ctaatgttgttgcagttcgctcgtagctgaattttgagctatatcgcggctcaacactgtaggtcatcgttggtagtgatgtcataat
cgactgtgcataaaccttgatgctcaaggtaggcctttatagggtagatacacagtgcatggcattgtggaacaaggcatctcgc
ggcttagttggtgggccgcgggggcaatgattaatagggaggagcgggggccttcatattgcagggcgagaggtgaaattc
gttgacccttgcaagatgtcctacagcgaaagcattggccaagtgcctccccattagtcaagaacgaaagtttggggatcaaag
acgatcagataccgtcgtagtccaaaccataaactatgtcgaccagcgattaggcgggctaccttcttcgagagctgcctagca
gcttgtgggaaaccatgagtgcttggactctgggggagtatggtcgcaaggctgaaacttaaaggaattgacggaagggc
acaccatggagtggagcctgcggcttaatttgactcaacacgggaaagcttaccaagctcagatatgataaggattgacagact
aaaagatctttcatgatctcataagtggtggtgcatggtcgttcttagttggtggagtgatttgtcaggtcaattccggtaacggac
gagacctcgacctgctaactagtgggattcattccttcgctcgacgaggcaggctttgctttgattatagggggcaacctttatagt
cgggtaaggtttgtattagtctgggggagtgggtttcatataattaatcttcctagaggtacttctggctctaagccagaggaagt
ccgaggcaataacaggtctgtgatgcccttagatatcttgggccgcacgcgtgctacaatgtaggcgctaatgagtctatttatcc
agctccgtaaggagtctggtaatcttggaatcccctgcgtgattgggcttgaccactgtaactgtggtcatcaacgaggaattcct
tgtatgcgcgagtcactatctcgcgcagaatctgtccctgccctttgtacacaccgcccgtcgctcctaccgatcgaacgatcagg
taaagtggacagagagttt--------------------------------
>KF662201.1_Cavenderia_aureostipes_OH396
----
tgtatcttccagggcatgtcgcaagaccttctggataaccgcagtaaatcggggctaatacatataaacggagggatggagag
ggcaaccttgaagtttctgcgatggacaattagttattcgaccaaccccgcaagggaatggttggaaccggttcatattgctaatc
gactctagcttgctagtagtctgataagctatagacaaccgccctatcaacttgatggtaaggttttggcttaccatggttgtaacg
ggtaacggggaatcagggttcgattccggagaggacgcctgagaaacggcgtccacatctacgggtggcagcaggcgcgt
aaattgcccaatctcaacagagaggaggcggtgacaataaatcccgatggctttgggggcaaccccaggctaatcagaataa
gtacacattaaatcccttaaccaatataattggagggcaagtctggtgccagcagccgcggtaattccagctccaatagcatata
ctaatgttgttgcagttcgctcgtagctgaattttgagctatatcgcggctcaacactgtaggtcatggctagcaatagttgcataat
cgactgtgcataaaccttgatgctcaaggtaggcctttatagggtagatacacagtgcatggcattgtggaacaaggcatctcgc
ggcttagttggtgggccgcgggggcaatgattaatagggaggagcgggggccttcatattgcagggcgagaggtgaaattc
gttgacccttgcaagatgtcctacagcgaaagcattggccaagtgcctccccattagtcaagaacgaaagtttggggatcaaag
acgatcagataccgtcgtagtccaaaccataaactatgtcgaccagcgattaggcgggctaccttcttcgagagctgcctagca

gcttgtgggaaaccatgagtgcttggactctggggggagtatggtcgcaaggctgaaacttaaaggaattgacggaagggc
acaccatggagtggagcctgcggcttaatttgactcaacacgggaaagcttaccaagctcagatatgattaggattgacagact
aaaagatctttcatgatctcataagtggtggtgcatggtcgttcttagttggtggagtgatttgtcaggtcaattccggtaacggac
gagacctcgacctgctaactagtgggattcattctttcgattgacgaggcaggtcttgctttgattatagggggcaacttctatagt
cgggtagggtttgtagtagtctggaggagtggatttcaaaaaattaatcttcctagaggtacttctggctctaagccagaggaag
tccgaggcaataacaggtctgtgatgcccttagatatcttgggccgcacgcgtgctacaatgtaggcgctaatgagtttttacatc
catctccgcaaggagtatggtaatcttggaatcacctgcgtgattgggcttgaccactgtaactgtggtcatcaacgaggaattcc
ttgtatgcgcgagtcactatctcgcgcagaatctgtccctgccctttgtacacaccgcccgtcgctcctaccgatcgaacgatcag
gtaaagtggacagagggtacttg------------------------------
>KF662199.1_Cavenderia_aureostipes_B15A
-
atgcatgtctaagtataagctcttgtacggctagactgcagacggctcattacaacggttgtatcttccagggcatgtcgcaagac
cttctggataaccgcagtaaatcggggctaatacatacaaacggagggatggagagggcaaccttgaagtttctgcgatggac
aattagttattcgaccaaccccgcaagggaacggttggaaccggttcatattgctaatcgactctagcttgctagtagtctgataa
gctatagacaaccgccctatcaacttgatggtaaggttttggcttaccatggttgtaacgggtaacggggaatcagggttcgatt
ccggagaggacgcctgagaaacggcgtccacatctacgggtggcagcaggcgcgtaaattgcccaatctcaacagagagg
aggcggtgacaataaatcccgatggctttgggggcaaccccaggctaatcagaataagtacacattaaatcccttaaccaatat
aattggagggcaagtctggtgccagcagccgcggtaattccagctccaatagcatatactaatgttgttgcagttcgctcgtagc
ttaattttgagctatatcgcggctcaacactgtaggtcatggctagcaatagttgcataatcgactgtgcataaaccttgatgctca
aggtaggcctttatagggtagatacacagtgcatggcattgtggaacaaggcatctcgcggcttagttggtgggccgcgggg
gcaatgattaatagggaggagcgggggccttcatattgcagggcgagaggtgaaattcgttgacccttgcaagatgtcctaca
gcgaaagcattggccaagtgcctccccattagtcaagaacgaaagtttggggatcaaagacgatcagataccgtcgtagtcca
aaccataaactatgtcgaccagcgattaggcgggctaccttcttcgagagctgcctagcagcttgtgggaaaccatgagtgctt
ggactctggggggagtatggtcgcaaggctgaaacttaaaggaattgacggaagggcacaccatggagtggagcctgcgg
cttaatttgactcaacacgggaaagcttaccaagctcagatatgattaggattgacagactaaaagatctttcatgatctcataagt
ggtggtgcatggtcgttcttagttggtggagtgatttgtcaggtcaattccggtaacggacgagacctcgacctgctaactagtg
ggattcattctttcgattgacgaggcaggtcttgctttgattatagggggcaacttttatagtcgggtacggtttgtagtagtctgg
aggagtggatttcaaaaaattaatcttcctagaggtacttctggctctaagccagaggaagtccgaggcaataacaggtctgtg
atgcccttagatatcttgggccgcacgcgtgctacaatgtaggcgctaatgagtttttacatccatctccgcaaggagtatggtaa
tcttggaatcacctgcgtgattgggcttgaccactgtaactgtggtcatcaacgaggaattccttgtatgcgcgagtcactatctcg
cgcagaatctgtccctgccctttgtacacaccgcccgtcgctcctaccgatcgaacgatcaggtaaagtggacagagggcactt
ggaagtccattgaacgccg-ttagaggaag
>GQ496157.1_Cavenderia_fasciculoidea
catgcatgtctaagtataagctcttgtacggctagactgcagacggctcattacaacggttgtatcttacagggcatgtcgcaaga
ccttctggataaccgcagtaaatcggggctaatacatataaacggagggatggagagggtaaccttgaagtttctgcgatgga
catttagctattcgaccaaccccgcaagggaatggttggaaccggttcatattgctaatcgactctagcttgctagtagtctgataa
gctatagacaaccgccctatcaacttgatggtaaggttttggcttaccatggttgtaacgggtaacggggaatcagggttcgatt
ccggagaggacgcctgagaaacggcgtccacatctacgggtggcagcaggcgcgtaaattgcccaatctcaacagagagg
aggcggtgacaataaatcccgatggctttgggggcaaccccaggctaatcagaataagtacacattaaatcccttaaccaatat
aattggagggcaagtctggtgccagcagccgcggtaattccagctccaatagcatatactaatgttgttgcagttcgctcgtagc
ttaattttgagctatatcgcggctcaacactgtaggtcatggctagcaatagttgcataatcgactgtgcataaaccttgatgctca
aggtaggcctttatagggtagatacacagtgcatggcattgtggaacaaggcatctcgcggcttagttggtgggccgcgggg
gcaatgattaatagggaggagcgggggccttcatattgcagggcgagaggtgaaattcgttgacccttgcaagatgtcctaca
gcgaaagcattggccaagtgcctccccattagtcaagaacgaaagtttggggatcaaagacgatcagataccgtcgtagtcca

aaccataaactatgtcgaccagcgattaggcgggctaccttcttcgagagctgcctagcagcttgtgggaaaccatgagtgctt
ggactctgggggagtatggtcgcaaggctgaaacttaaaggaattgacggaagggcacaccatggagtggagcctgcgg
cttaatttgactcaacacgggaaagcttaccaagctcagatatgattaggattgacagactaaaagatctttcatgatctcataagt
ggtggtgcatggtcgttcttagttggtggagtgatttgtcaggtcaattccggtaacggacgagacctcgacctgctaactagtg
ggattcattctttcgattgacgaggcaggtattgctttgattatagggggcaacttttatagtcgggtagtgcttgtattagtctggg
agagtgggtttcaaataattaatcttcctagaggtacttctggctctaagccagaggaagtccgaggcaataacaggtctgtgat
gcccttagatatcttgggccgcacgcgtgctacaatgtaggcgctaatgagtttttacatccatctccgcaaggagtatggtaatct
tggaatcccctgcgtgattgggcttgaccactgtaactgtggtcatcaacgaggaattccttgtatgcgcgagtcactatctcgcg
cagaatctgtccctgccctttgtacacaccgcccgtcgctcctaccgatcgaacgatcaggtaaagtggacagagggttgttgga
agtccattgaacgccg-ttagaggaag
>OM677259.1_Cavenderia_ungulata_TH18B
----
aggtataagctcttgtacggctagactgcagacggctcattacaacggttgtatcttccagggcatgtcgcaagaccttctggata
accgcagtaaatcggggctaatacatacaaacggagggatggagagggcaaccttgaagtttctgcgatggacaattagttat
tcgaccaaccccgcaagggaatggttggaaccggttcatattgctaatcgactctagcttgctagtagtctgataagctatagac
aaccgccctatcaacttgatggtaaggttttggcttaccatggttgtaacgggtaacggggaatcagggttcgattccggagag
gacgcctgagaaacggcgtccacatctacgggtggcagcaggcgcgtaaattgcccaatctcaacagagaggaggcggtga
caataaatcccgatggctttgggggcaaccccaggctaatcagaataagtacacattaaatcccttaaccaatataattggaggg
caagtctggtgccagcagccgcggtaattccagctccaatagcatatactaatgttgttgcagttcgctcgtagcttaattttgagc
tatatcgcggctcaacactgtaggtcatggctagcaatagttgcataatcgactgtgcataaaccttgatgctcaaggtaggcctt
tatagggtagatacacagtgcatggcattgtggaacaaggcatctcgcggcttagttggtgggccgcgggggcaatgattaat
agggaggagcgggggccttcatattgcagggcgagaggtgaaattcgttgacccttgcaagatgtcctacagcgaaagcatt
ggccaagtgcctccccattagtcaagaacgaaagtttggggatcaaagacgatcagataccgtcgtagtccaaaccataaacta
tgtcgaccagcgattaggcgggctaccttcttcgagagctgcctagcagcttgtgggaaaccatgagtgcttggactctgggg
ggagtatggtcgcaaggctgaaacttaaaggaattgacggaagggcacaccatggagtggagcctgcggcttaatttgactc
aacacgggaaagcttaccaagctcagatatgattaggattgacagactaaaagatctttcatgatctcataagtggtggtgcatg
gtcgttcttagttggtggagtgatttgtcaggtcaattccggtaacggacgagacctcgacctgctaactagtgggattcattcttt
cgattgacgaggcaggtcttgctttgattatagggggcaacttctatagtcgggtacggtttgtagtagtctggaggagtggatt
tcaaaaaattaatcttcctagaggtacttctggctctaagccagaggaagtccgaggcaataacaggtctgtgatgcccttagat
atcttgggccgcacgcgtgctacaatgtaggcgctaatgagtttttacatccatctccgcaaggagtatggtaatcttggaatcac
ctgcgtgattgggcttgaccactgtaactgtggtcatcaacgaggaattccttgtatgcgcgagtcactatctcgcgcagaatctg
tccctgccctttgtacacaccgcccgtcgctcctaccgatcgaacgatcaggtaaagtggacagagggttcttggaagtccattg
aacgccg-ttagaggaag
>OM677258.1_Cavenderia_protumula_TH20A
catgcatgtctaagtataagctcttgtacggctagactgcagacggctcattacaacggttgtatcttccagggcatgtcgcaaga
ccttctggataaccgcagtaaatcggggctaatacatacaaacggagggatggagagggcaaccttgaagtttctgcgatgga
caattagttattcgaccaaccccgcaagggaatggttggaaccggttcatattgctaatcgactctagcttgctagtagtctgataa
gctatagacaaccgccctatcaacttgatggtaaggttttggcttaccatggttgtaacgggtaacggggaatcagggttcgatt
ccggagaggacgcctgagaaacggcgtccacatctacgggtggcagcaggcgcgtaaattgcccaatctcaacagagagg
aggcggtgacaataaatcccgatggctttgggggcaaccccaggctaatcagaataagtacacattaaatcccttaaccaatat
aattggagggcaagtctggtgccagcagccgcggtaattccagctccaatagcatatactaatgttgttgcagttcgctcgtagc
ttaattttgagctatatcgcggctcaacactgtaggtcatggctagcaatagttgcataatcgactgtgcataaaccttgatgctca
aggtaggcctttatagggtagatacacagtgcatggcattgtggaacaaggcatctcgcggcttagttggtgggccgcgggg
gcaatgattaatagggaggagcgggggccttcatattgcagggcgagaggtgaaattcgttgacccttgcaagatgtcctaca

gcgaaagcattggccaagtgcctccccattagtcaagaacgaaagtttggggatcaaagacgatcagataccgtcgtagtcca
aaccataaactatgtcgaccagcgattaggcgggctaccttcttcgagagctgcctagcagcttgtgggaaaccatgagtgctt
ggactctgggggggagtatggtcgcaaggctgaaacttaaaggaattgacggaagggcacaccatggagtggagcctgcgg
cttaatttgactcaacacgggaaagcttaccaagctcagatatgattaggattgacagactaaaagatctttcatgatctcataagt
ggtggtgcatggtcgttcttagttggtggagtgatttgtcaggtcaattccggtaacggacgagacctcgacctgctaactagtg
ggattcattctttcgattgacgaggcaggtcttgctttgattataggggggcaacttctatagtcgggtacggtttgtagtagtctgg
aggagtggatttcaaaaaattaatcttcctagaggtacttctggctctaagccagaggaagtccgaggcaataacaggtctgtg
atgcccttagatatcttgggccgcacgcgtgctacaatgtaggcgctaatgagtttttacatccatctccgcaaggagtatggtaa
tcttggaatcacctgcgtgattgggcttgaccactgtaactgtggtcatcaacgaggaattccttgtatgcgcgagtcactatctcg
cgcagaatctgtccctgccctttgtacacaccgcccgtcgctcctaccgatcgaacgatcaggtaaagtggacagagggttcttg
gaagtccattgaacgccg-ttagaggaag
>OM677256.1_Cavenderia_parvibrachiata_TH20C
-------------gaagaaacctttgc------------
cagacgggctttcacaacggttgtatcttccagggcatgtcgcaagaccttctggataaccgcagtaaatcggggctaatacata
caaacggagggatggagagggcaaccttgaagtttctgcgatggacaattagttattcgaccaaccccgcaagggaatggtt
ggaaccggttcatattgctaatcgactctagcttgctagtagtctgataagctatagacaaccgccctatcaacttgatggtaaggt
tttggcttaccatggttgtaacgggtaacggggaatcagggttcgattccggagaggacgcctgagaaacggcgtccacatct
acgggtggcagcaggcgcgtaaattgcccaatctcaacagagaggaggcggtgacaataaatcccgatggctttgggggca
accccaggctaatcagaataagtacacattaaatcccttaaccaatataattggagggcaagtctggtgccagcagccgcggta
attccagctccaatagcatatactaatgttgttgcagttcgctcgtagcttaattttgagctatatcgcggctcaacactgtaggtcat
ggctagcaatagttgcataatcgactgtgcataaaccttgatgctcaaggtaggcctttatagggtagatacacagtgcatggca
ttgtggaacaaggcatctcgcggcttagttggtgggccgcgggggcaatgattaatagggaggagcgggggccttcatattg
cagggcgagaggtgaaattcgttgacccttgcaagatgtcctacagcgaaagcattggccaagtgcctccccattagtcaaga
acgaaagtttggggatcaaagacgatcagataccgtcgtagtccaaaccataaactatgtcgaccagcgattaggcgggctac
cttcttcgagagctgcctagcagcttgtgggaaaccatgagtgcttggactctgggggggagtatggtcgcaaggctgaaactta
aaggaattgacggaagggcacaccatggagtggagcctgcggcttaatttgactcaacacgggaaagcttaccaagctcaga
tatgattaggattgacagactaaaagatctttcatgatctcataagtggtggtgcatggtcgttcttagttggtggagtgatttgtca
ggtcaattccggtaacggacgagacctcgacctgctaactagtgggattcattctttcgattgacgaggcaggtcttgctttgatt
ataggggggcaacttctatagtcgggtacggtttgtagtagtctggaggagtggatttcaaaaaattaatcttcctagaggtacttc
tggctctaagccagaggaagtccgaggcaataacaggtctgtgatgcccttagatatcttgggccgcacgcgtgctacaatgta
ggcgctaatgagtttttacatccgtctccgcaaggagtacggtaatcttggaatcacctgcgtgattgggcttgaccactgtaact
gtggtcatcaacgaggaattccttgtatgcgcgagtcactatctcgcgcagaatctgtccctgccctttgtacacaccgcccgtcg
ctcctaccgatcgaacgatcaggtaaagtggacagagggttcttggaagtccattgaacgccgtttagaggaag
>OM677257.1_Cavenderia_parvibrachiata_2019TH20C
catgcatgtctaagtataagctcttgtacggctagactgcagacggctcattacaacggttgtatcttccagggcatgtcgcaaga
ccttctggataaccgcagtaaatcggggctaatacatacaaacggagggatggagagggcaaccttgaagtttctgcgatgga
caattagttattcgaccaaccccgcaagggaatggttggaaccggttcatattgctaatcgactctagcttgctagtagtctgataa
gctatagacaaccgccctatcaacttgatggtaaggttttggcttaccatggttgtaacgggtaacggggaatcagggttcgatt
ccggagaggacgcctgagaaacggcgtccacatctacgggtggcagcaggcgcgtaaattgcccaatctcaacagagagg
aggcggtgacaataaatcccgatggctttgggggcaaccccaggctaatcagaataagtacacattaaatcccttaaccaatat
aattggagggcaagtctggtgccagcagccgcggtaattccagctccaatagcatatactaatgttgttgcagttcgctcgtagc
ttaattttgagctatatcgcggctcaacactgtaggtcatggctagcaatagttgcataatcgactgtgcataaaccttgatgctca
aggtaggcctttatagggtagatacacagtgcatggcattgtggaacaaggcatctcgcggcttagttggtgggccgcgggg
gcaatgattaatagggaggagcgggggccttcatattgcagggcgagaggtgaaattcgttgacccttgcaagatgtcctaca

gcgaaagcattggccaagtgcctccccattagtcaagaacgaaagtttggggatcaaagacgatcagataccgtcgtagtcca
aaccataaactatgtcgaccagcgattaggcgggctaccttcttcgagagctgcctagcagcttgtgggaaaccatgagtgctt
ggactctgggggagtatggtcgcaaggctgaaacttaaaggaattgacggaagggcacaccatggagtggagcctgcgg
cttaatttgactcaacacgggaaagcttaccaagctcagatatgattaggattgacagactaaaagatctttcatgatctcataagt
ggtggtgcatggtcgttcttagttggtggagtgatttgtcaggtcaattccggtaacggacgagacctcgacctgctaactagtg
ggattcattctttcgattgacgaggcaggtcttgctttgattataggggcaacttctatagtcgggtacggtttgtagtagtctgg
aggagtggatttcaaaaaattaatcttcctagaggtacttctggctctaagccagaggaagtccgaggcaataacaggtctgtg
atgcccttagatatcttgggccgcacgcgtgctacaatgtaggcgctaatgagtttttacatccgtctccgcaaggagtacggtaa
tcttggaatcacctgcgtgattgggcttgaccactgtaactgtggtcatcaacgaggaattccttgtatgcgcgagtcactatctcg
cgcagaatctgtccctgccctttgtacacaccgcccgtcgctcctaccgatcgaacgatcaggtaaagtggacagagggttcttg
gaagtccattgaacgccgtttagagggaa
>OM677255.1_Cavenderia_helicoidea_TH19B
catgcatgtctaagtataagctcttgtacggctagactgcagacggctcattacaacggttgtatcttccagggcatgtcgcaaga
ccttctggataaccgcagtaaatcggggctaatacatacaaacggagggatggagagggcaaccttgaagtttctgcgatgga
caattagttattcgaccaaccccgcaagggaatggttggaaccggttcatattgctaatcgactctagcttgctagtagtctgataa
gctatagacaaccgccctatcaacttgatggtaaggttttggcttaccatggttgtaacgggtaacggggaatcagggttcgatt
ccggagaggacgcctgagaaacggcgtccacatctacgggtggcagcaggcgcgtaaattgcccaatctcaacagagagg
aggcggtgacaataaatcccgatggctttgggggcaaccccaggctaatcagaataagtacacattaaatcccttaaccaatat
aattggagggcaagtctggtgccagcagccgcggtaattccagctccaatagcatatactaatgttgttgcagttcgctcgtagc
ttaattttgagctatatcgcggctcaacactgtaggtcatggctagcaatagttgcataatcgactgtgcataaaccttgatgctca
aggtaggcctttatagggtagatacacagtgcatggcattgtggaacaaggcatctcgcggcttagttggtgggccgcgggg
gcaatgattaatagggaggagcgggggccttcatattgcagggcgagaggtgaaattcgttgacccttgcaagatgtcctaca
gcgaaagcattggccaagtgcctccccattagtcaagaacgaaagtttggggatcaaagacgatcagataccgtcgtagtcca
aaccataaactatgtcgaccagcgattaggcgggctaccttcttcgagagctgcctagcagcttgtgggaaaccatgagtgctt
ggactctgggggagtatggtcgcaaggctgaaactaaaaggaattgacggaagggcacaccatggagtggagcctgcgg
cttaatttgactcaacacgggaaagcttaccaagctcagatatgattaggattgacagactaaaagatctttcatgatctcataagt
ggtggtgcatggtcgttcttagttggtggagtgatttgtcaggtcaattccggtaacggacgagacctcgacctgctaactagtg
ggattcattctttcgattgacgaggcaggtcttgctttgattataggggcaacttctatagtcgggtacggtttgtagtagtctgg
aggagtggatttcaaaaaattaatcttcctagaggtacttctggctctaagccagaggaagtccgaggcaataacaggtctgtg
atgcccttagatatcttgggccgcacgcgtgctacaatgtaggcgctaatgagtttttacatccatctccgcaaggagtatggtaa
tcttggaatcacctgcgtgattgggcttgaccactgtaactgtggtcatcaacgaggaattccttgtatgcgcgagtcactatctcg
cgcagaatctgtccctgccctttgtacacaccgcccgtcgctcctaccgatcgaacgatcaggtaaagtggacagagggttcttg
gaagtccattgaacgccg-ttagagaagg
>X13160.1_Physarum_polycephalum
catgcatgtctaagagcaagtctctct-----
gaatctgcgaacggctccgcataccagttgtaaaccatagcaagcgccgcaaggccacagggataaccctggtaattcgagg
ctaatacataccaccgcttcgacccgtaaggggagggcgggggttgtgtgacccaggtcgcaaatattaactgggagtg---
------
gcacacgatctgaccaccataccaaacggttatccgcttcgaaagcttcggtgagctgtgtgcttctgacctatcaactagatggc
agcgtaacggcatgccatggtaacaacgggt-
acagaggataagggttcgatcctggagagtgggcctgagagattgctcacacttctaaggaaggcagcaggcgcgcaacgt
tcccattgggcaaagctcgagggcgttag-gggacatatgaatgcctgc-----
cttatggtgggcaattcaaatgggactgttttaaacatcctatcgagtacaattagaggacaagtctggtgccagcacccgcggt
aattccagctctaatagcatacgttaaagttgttgcggttcgctcgtagtcggcttcagagcctggtcagtgcccgagtcacagg

gtcgtcgatcgcggggctcgtaccaatcaccatgattaaaccgtagtgaccaaagcacgtc--
tttagacgggcacggcacagcatgggac---
gaaacgcaccggctcgccttttttttgcggggcgtgactcggtaaaagcgaaagggatgttcgagggtgaccgaattgctggg
cgagtggtgaaatacgttgaccctagcaagtcgaccaaaggcgtaagcagtcatcaagggcattcccgttgatcaagagcga
aagttaagggttcgaagacgatcagataccgtcgtagtcttaactataaatgatgcagaccagggataggaca----
gtgtccatctcgactcttccgg--
accttggagaaatcacgagtctatgggttctgggggagtatggtcgcaaggctgaaacttaaaggaattgacggaagggca
ca-
caaagagtggaacctgcggcttaatttgactcaacacgggaaaactcaccaggtccggatacacgtatgaaagtcaagctgaa
agactttactcaatgatgtaagtggtggtgcatggtcgttcttagttcgtggattgatttgtctggtttattccgataacgagcgaga
ccccggcgttcctaataggggtggcagccagaccgtcgcaagacaggttagctcg--------------------------
------------------ccacctg-----------------
aagttatgcttcttagacgtatcagagccgataaggttcttgaaat--
gggttaataacaggtcagtcatgcccttagatgttctgggccgcacgcgcgttacaatggcatgtaaaacgagtgacaaggcg
tcccacggccgaaggtcgtggtaacccttagtccctgctttgactgggacagatctttgcaattatggtctcaaacgaggaattttt
agtaatcgcaggtcattaacctgcgttgaatgcgtccctgccctttgtacacaccgcccgtcgctgctaccgattgggttttacagtt
acgcgttcggagggctccggggcgtcggccaaatacggcctagaggaag
